# Hydrogen peroxide from l-amino acid oxidase of king cobra (*Ophiophagus hannah*) venom attenuates *Pseudomonas* biofilms

**DOI:** 10.1038/s41598-023-37914-3

**Published:** 2023-07-12

**Authors:** Uthaibhorn Singkham-In, Wichit Thaveekarn, Jureeporn Noiphrom, Orawan Khow, Surada Ponwaranon, Jiraphorn Issara-Amphorn, Visith Sitprija, Asada Leelahavanichkul

**Affiliations:** 1 Department of Microbiology, Faculty of Medicine, Chulalongkorn University, 1873 Rama 4 Road, Pathumwan, Bangkok, 10330 Thailand; 2 Center of Excellence in Translational Research in Inflammatory and Immunology (CETRII), Faculty of Medicine, Chulalongkorn University, 1873 Rama 4 Road, Pathumwan, Bangkok, 10330 Thailand; 3 Queen Saovabha Memorial Institute, Thai Red Cross Society, 1871 Rama 4 Road, Pathumwan, Bangkok, 10330 Thailand; 4 Functional Cellular Networks Section, Laboratory of Immune System Biology, National Institute of Allergy and Infectious Diseases, National Institutes of Health, Bethesda, MD 20892 USA

**Keywords:** Microbiology, Molecular biology, Medical research

## Abstract

Because of the high incidence of *Pseudomonas aeruginosa* biofilms-related nosocomial infections, venoms from common Thai snakes were tested. Although venoms from king cobra (*Ophiophagus hannah*; OH) and green pit viper (*Trimeresurus albolabris*) showed the broadest antibacterial spectrum, OH venom demonstrated more profound anti-biofilm activities against *P. aeruginosa*. Additionally, purified l-amino acid oxidase from OH venom (OH-LAAO), using a three-step chromatography and protein identification, reduced biofilm mass as indicated by the downregulation of several genes, including the genes for biofilm synthesis (*algD* and *pslB*) and biofilm regulators (*algU*, *gacA*, and *siaD*). Moreover, OH-LAAO disrupted *Pseudomonas*-preformed biofilms via upregulation of several genes for biofilm dispersion (*nbdA*, *bdlA*, and *dipA*) and biofilm degradation (*endA* and *pslG*), resulting in a reduction of the biofilm biomass. Due to the antimicrobial effects and anti-biofilm activities (reduced production plus increased dispersion) neutralized by catalase, a hydrogen peroxide (H_2_O_2_)-degrading enzyme, the enhanced H_2_O_2_ by OH venom might be one of the anti-biofilm mechanisms. Hence, OH-LAAO was proposed as a novel agent against *Pseudomonas* biofilms for either treatment or prevention. More studies are interesting.

## Introduction

The venomous snake, one of the most dangerous animals, is widely found in the world, especially in tropical regions^1^. Among all, the most venomous snakes are Elapidae (elapids) and Viperidae (viperids or vipers), which produce neurotoxins and myotoxic agents, respectively^2^. As such, the Elapids, such as *Naja kaouthia* (NK; cobra) and *Ophiophagus hannah* (OH; king cobra), have permanent front fangs, whereas the vipers, including *Dabonia russellii* (DR; Russell's viper) and *Trimeresurus albolabris* (TA; green pit viper), have retractable front fangs for venom ejection. While snake venoms contain numerous bioactive proteins that exhibit cytotoxicity toward several cells, including neuron cells, hematological cells, and myocytes^2^, several molecules from snake venoms are the new candidates for modern medicine treatment^3^. For example, antihypertensive bradykinin (vasodilator peptide) from South American pit viper^4^, anti-fibrinogen (thrombin-like serine protease) for ischemic stroke from Brazilian pit viper^5,6^, and anti-platelet from three-finger toxin (3FTx) of Indian cobra^7^. For antimicrobial activity, various proteinaceous compounds in snake venoms are antimicrobials^2^ against multiple organisms (*Pseudomonas aeruginosa, Candida albicans,* and *Staphylococcus aureus*) through phospholipase A2 (PLA2) and l-amino acid oxidase (LAAO) from Lebanon viper ^8^ and antimicrobial peptide (Cathelicidin) for multidrug-resistant *Acinetobacter baumannii* and methicillin-resistant *S. aureus* (MRSA) from king cobra snake^9^. Biofilms are the alteration from a free-living (planktonic) form of bacteria into a sessile form with self-secreted exopolysaccharide (EPS) protecting organisms from most of the antimicrobial agents as one of the antibiotic resistance mechanisms^10^. Interestingly, there are several anti-biofilms from snake venoms^11,12^, including the prevention of *A. baumannii* biofilms by Caatinga lancehead snake-isolated PLA2^12^, and *S. aureus* biofilm eradication by C-type lectin purified from *Bothrops jararacussu* (a South American pit viper)^11^. However, data on the anti-biofilms from snake venoms are still very less, especially against *P. aeruginosa*.

Biofilms from either Gram-positive or Gram-negative bacteria induce antibiotic resistance, and biofilm prevention and eradication are clinically useful, especially in patients with indwelling catheters^13,14^. Although anti-biofilms toward multi-organisms are easy for the clinical use, anti-biofilms against an individual organism demonstrated clearer mechanistic actions due to the different mechanisms of biofilm production for different microbes. Because *P. aeruginosa* infection is an emerging health-care problem among several biofilm-producing pathogens, partly due to the effective biofilm formation in several strains, that is demonstrated in several chronic infections (cystic fibrosis, catheter-induced infection, and chronic wounds)^15^, anti-*Pseudomonas* biofilms might be beneficial. The exopolysaccharides (EPS) of *Pseudomonas* biofilms consist of alginate, d-mannuronic acid, l-guluronic acid polysaccharide, and PSL which is a neutral pentasaccharide subunit containing mannose, rhamnose, and glucose controlled by the polysaccharide synthesis locus (*psl*) gene ^16^. The embedment of sessile-formed bacteria into these biofilm substances is responsible for antimicrobial resistance, despite the in vitro susceptibility to antibiotics in the planktonic bacterial form^17^. In patients with retained catheters (urinary, venous, peritoneal, and hemodialysis catheters), biofilms cause several episodes of infection with increased morbidity and mortality^13,18^. Because of profound polysaccharide components in biofilms and the possible carbohydrate digestibility of enzymes, surface-bound glycoside hydrolase enzymes prevent PSL attachment on the catheter surface and are effective for *Pseudomonas* biofilm prevention^19^. However, snake venoms are fascinating sources of powerful enzymes that might act on the mature biofilms, especially for catheter lock solutions in several indwelling catheters^20^ and adjunctive antimicrobial agents.

Here, we evaluated the anti-biofilm effect of snake venoms commonly found in Thailand and identified the possible effective enzyme against *Pseudomonas* biofilms that might be clinically beneficial.

## Results

### Antimicrobial properties of crude snake venoms, especially *O. hannah* (king cobra) and *T. albolabris* (green pit viper), as a broad-spectrum antimicrobial

Because the antimicrobial effect plays a crucial role in biofilm eradication (eradication of the dispersed cells), we screened antimicrobial properties of the venoms from Thai common snakes, including *Naja kaouthia* (NK; cobra), *Ophiophagus hannah* (OH; king cobra), *Dabonia russellii* (DR; Russell’s viper), and *Trimeresurus albolabris* (TA; green pit viper). As such, the in vitro antimicrobial activities, including the minimum inhibitory concentrations (MICs) and the minimum bactericidal concentrations (MBCs), were performed against Gram-negative and Gram-positive bacteria. The maximum concentration of crude venoms was 0.25 mg/mL to avoid the profound dilution of bacterial media (too much dilution of bacterial broth) (see “Methods” Section). As such, OH and TA venoms exhibited bacterial inhibitions against all selected *P. aeruginosa* isolates (MIC range between 0.06 and 0.25 mg/mL) (Table 1). In contrast, most venoms did not inhibit *Escherichia coli* ATCC 25922 (MIC > 0.25 mg/mL). Among Gram-positive bacteria, the growth of most *S. aureus* strains was interfered with all four venoms (MIC range 0.0038 to 0.25 mg/mL). All selected snake venoms did not affect *Enterococcus faecalis* (MIC > 0.25 mg/mL), and only OH and TA venoms had bactericidal activities against *P. aeruginosa* (MBC range 0.06 to 0.25 mg/mL). Additionally, all venoms exhibited bactericidal effects on *S. aureus* (MBC range 0.0038 to 0.25 mg/mL). Therefore, most snake venoms were effective against *S. aureus.* The venoms from OH and TA were categorized as broad-spectrum antimicrobial agents against both Gram-positive (*S. aureus*) and Gram-negative bacteria (*P. aeruginosa*).

**Table 1:** Antimicrobial, anti-biofilm, and biofilm eradication activities of crude snake venoms, including *Naja kaouthia* (NK), *Ophiophagus hannah* (OH), *Dabonia russellii* (DR), *Trimeresurus albolabris* (TA) venoms.

Bacterial strain	NK venom (mg/mL)				OH venom (mg/mL)				DR venom (mg/mL)				TA venom (mg/mL)			
	MIC	MBC	MBIC	MBEC	MIC	MBC	MBIC	MBEC	MIC	MBC	MBIC	MBEC	MIC	MBC	MBIC	MBEC
*P. aeruginosa* ATCC 27853	> 0.25	> 0.25	> 0.25	> 0.25	0.125	0.25	0.125	> 0.25	> 0.25	> 0.25	> 0.25	> 0.25	0.125	0.125	0.125	> 0.25
*P. aeruginosa* PAO1	> 0.25	> 0.25	> 0.25	> 0.25	0.25	0.25	0.25	> 0.25	> 0.25	> 0.25	> 0.25	> 0.25	0.25	0.25	0.125	> 0.25
*P. aeruginosa* PACL	> 0.25	> 0.25	> 0.25	> 0.25	0.25	0.25	0.25	> 0.25	> 0.25	> 0.25	> 0.25	> 0.25	0.06	0.125	0.125	> 0.25
*P. aeruginosa* C_PACL	> 0.25	> 0.25	> 0.25	> 0.25	0.06	0.125	0.125	> 0.25	> 0.25	> 0.25	> 0.25	> 0.25	0.06	0.06	0.06	> 0.25
*E. coli* ATCC 25922	> 0.25	> 0.25	> 0.25	> 0.25	> 0.25	> 0.25	> 0.25	> 0.25	> 0.25	> 0.25	> 0.25	> 0.25	> 0.25	> 0.25	> 0.25	> 0.25
*S. aureus* ATCC 29213	0.03	0.125	0.03	> 0.25	0.0038	0.075	0.0038	> 0.25	0.06	0.25	0.06	> 0.25	0.0038	0.0038	0.0038	> 0.25
*S. aureus* SA1	0.125	0.25	ND	ND	0.015	0.06	ND	ND	0.25	0.25	ND	ND	0.015	0.06	ND	ND
*S. aureus* SA2	0.03	0.03	ND	ND	0.0038	0.0038	ND	ND	0.06	0.06	ND	ND	0.0038	0.0038	ND	ND
*S. aureus* SA3	0.03	0.03	ND	ND	0.0038	0.0038	ND	ND	0.06	0.06	ND	ND	0.0038	0.0038	ND	ND
*E. faecalis* EF1	> 0.25	> 0.25	> 0.25	> 0.25	> 0.25	> 0.25	> 0.25	> 0.25	> 0.25	> 0.25	> 0.25	> 0.25	> 0.25	> 0.25	> 0.25	> 0.25
*E. faecalis* EF2	> 0.25	> 0.25	> 0.25	> 0.25	> 0.25	> 0.25	> 0.25	> 0.25	> 0.25	> 0.25	> 0.25	> 0.25	> 0.25	> 0.25	> 0.25	> 0.25
*E. faecalis* EF3	> 0.25	> 0.25	> 0.25	> 0.25	> 0.25	> 0.25	> 0.25	> 0.25	> 0.25	> 0.25	> 0.25	> 0.25	> 0.25	> 0.25	> 0.25	> 0.25

The minimum inhibitory concentrations (MICs) and the minimum bactericidal concentrations (MBCs) were determined for antimicrobials. The minimum biofilm inhibitory concentrations (MBICs) was performed for anti-biofilm activity. The minimum biofilm eradication concentrations (MBECs) was determined for biofilm eradication activity.

‘ND: Not determined (no biofilm production from *S*. *aureus* SA1, SA2, and SA3); all of the strains are clinical isolates, except for the ATCC strains and PAO1 (comercially available strains); PACL is a clinical-isolated *P*. *aeruginosa* that can produce more biofilms after stimulated by chlorhexidine (an-antiseptic) referred to as “chlorhexidine-treated *P*. *aeruginosa* (C_PACL)”.

### Crude snake venoms exhibited anti-biofilm activity and partially destroyed the established biofilms

Due to the effect on both Gram-positive and Gram-negative bacteria, OH and TA venoms were further tested for anti-biofilm activities (inhibition of biofilm formation) as determined by the minimum biofilm inhibitory concentrations (MBICs). As such, OH and TA venoms at the MIC (1 × MIC) or twofold higher (2 × MIC), identical to the MBIC range between 0.06 to 0.25 mg/mL, were adequate to prevent *Pseudomonas* biofilm (incubated venoms together with *P. aeruginosa*) (Table 1). Moreover, OH and TA venoms inhibited the biofilms of *S. aureus* ATCC 29213 (MBIC = 0.0038 mg/mL). Notably, the venoms without the antimicrobial effect did not exhibit anti-biofilm formation, especially in *E. coli* ATCC 25922 and three *E. faecalis* isolates (Table 1). Therefore, the biofilm prevention effect of OH and TA venoms against *P. aeruginosa* was mainly due to antimicrobial activities. Subsequently, to test the ability of snake venoms to eliminate biofilms (administration of venoms onto the preformed biofilms) as indicated by the minimum biofilm eradication concentrations (MBECs) were determined using bacterial-preformed biofilms in 96-well culture plates. Although all venoms had no biofilm eradication effect at the MBECs higher than 0.25 mg/mL, OH venom prominently decreased preformed biofilms from all *P. aeruginosa* isolates, including *P. aeruginosa* ATCC 27853 and *P. aeruginosa* strain PAO1 (PAO1) (the standard strains used for biofilm studies) together with the clinical-isolated *P. aeruginosa* (PACL) and chlorhexidine-treated clinical isolated *P. aeruginosa* (C_PACL) (Supplementary Figs. S1–S4). In contrast, there was no biofilm disruption effect of TA venom against the preformed biofilm of these *Pseudomonas* spp. (Supplementary Figs. S2–S4). Because OH venom was the most effective antimicrobial and anti-biofilm against *P. aeruginosa*, OH venom was further investigated for mechanistic experiments.

### King cobra venom (*O. hannah*) reduced *P. aeruginosa* extracellular polysaccharides, (alginate and PSL), partly through the regulation on *algU*/*mucA* and *gacA.*

**Figure 1:**
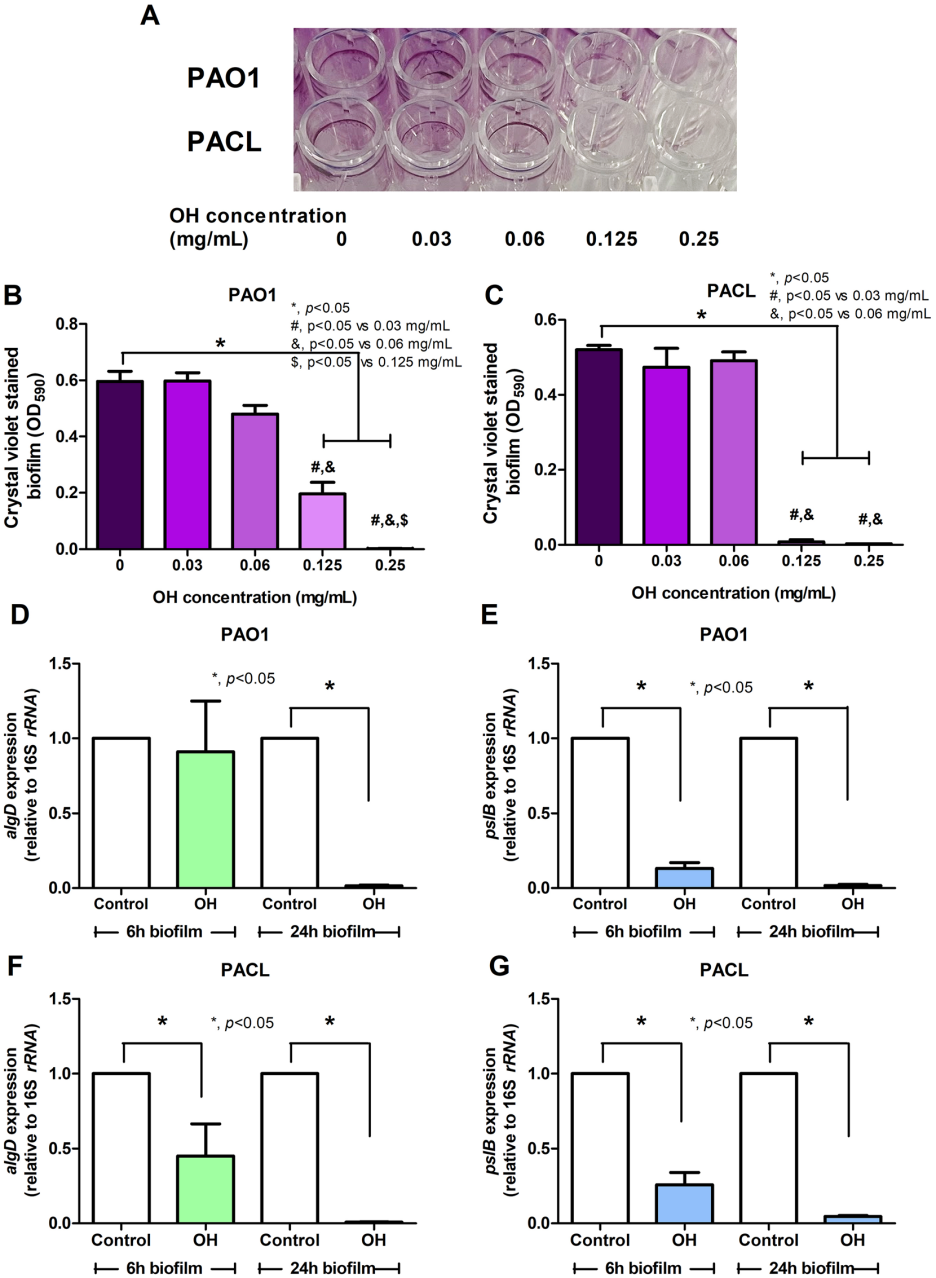
Anti-biofilm activities of crude king cobra venom (OH) against *P. aeruginosa* reference strain (PAO1) and clinical strain (PACL). The intensities of crystal violet stain from 24 h biofilms of *P. aeruginosa* PAO1 and clinically isolated strain (PACL) cultured with crude OH venoms with representative biofilm pictures in 96-well polystyrene plates stained with crystal violet are demonstrated (**A**–**C**). The expressions of genes encoding for alginate and PSL synthesis (**D**–**G**) including *algD* (**D**,**F**) and *pslB* (**E**,**G**), as determined in 6 h and 24 h biofilms of *P. aeruginosa* cultured with crude OH venom (at sub-MIC level, avoiding the lethal effect of the venom) by qRT-PCR, are demonstrated. The experiments were performed in independent triplicate. Mean ± SEM is presented with the paired *t-*test analysis (*p ˂ 0.05).

**Figure 2:**
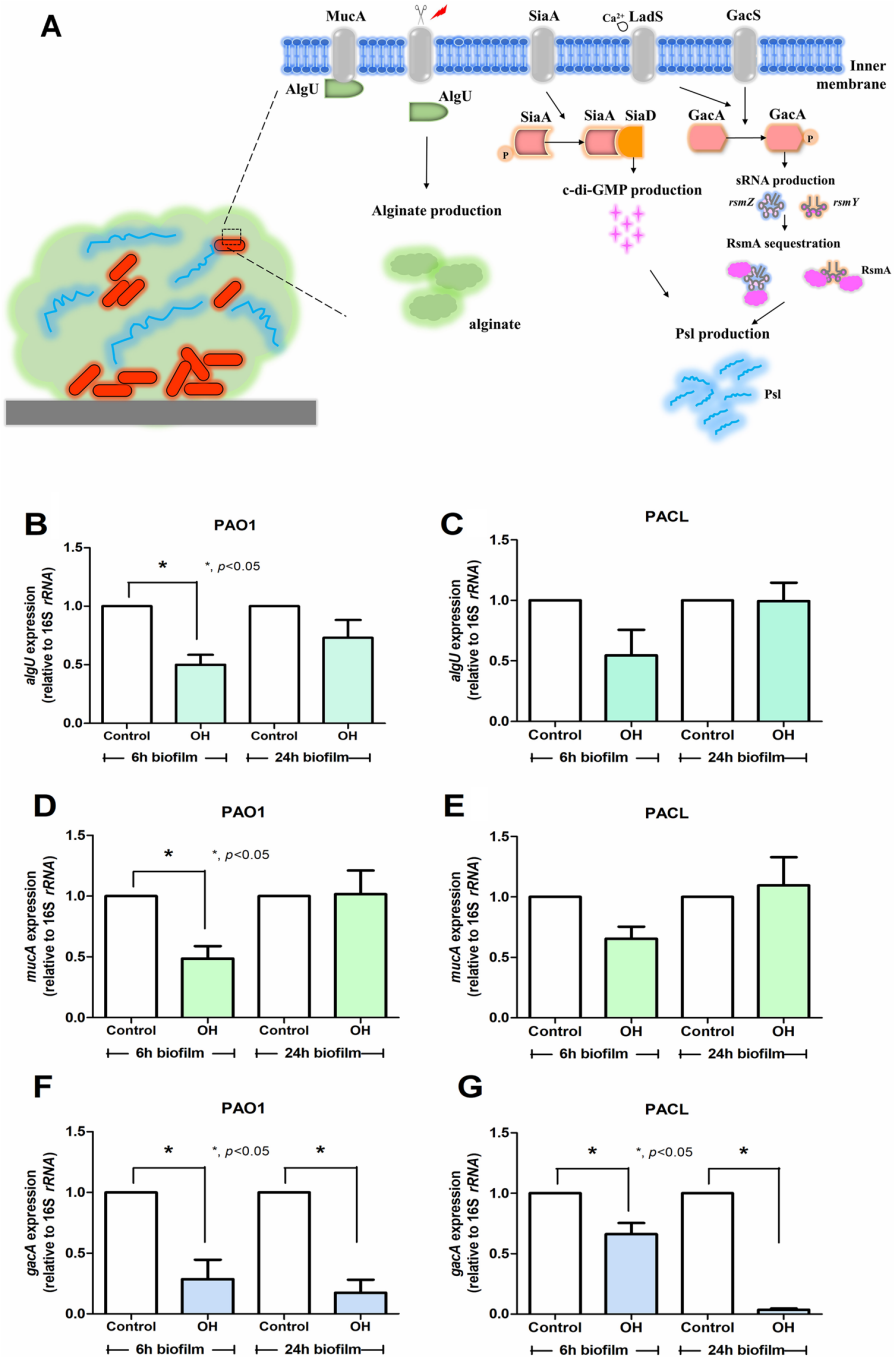
Crude king cobra venom (OH) reduced the production of alginate and PSL of *P. aeruginosa* reference strain (PAO1) and clinical strain (PACL) through the down-regulation of several genes. Diagram of extracellular polysaccharide (EPS, including alginate and PSL) productions (**A**) in *P. aeruginosa*, including (i) MucA inactivation by external stress resulting in the release of free sigma factor AlgU to switch on alginate synthesis operon, (ii) c-di-GMP-dependent PSL synthesis via activation of a diguanylate cyclase (SiaD) and c-di-GMP-independent pathway using *rsmZ* (regulated by GacA regulator). Gene expression profiles in *P. aeruginosa* PAO1 and *P. aeruginosa* clinical isolated strain (PACL) biofilms cultured with crude OH venom (at sub-MIC level, avoiding lethal effect of the venom) in alginate production, including *algU* (**B**,**C**) and *mucA* (**D**,**E**), and PSL production (*gacA*) (**F**,**G**) as determined by qRT-PCR are demonstrated (the experiments were performed in independent triplicate). Mean ± SEM is presented with the paired *t-*test analysis (*p˂0.05).

Because *P. aeruginosa* PAO1 (a standard strain) and PACL (a clinically isolated strain) prominently produced biofilms than other selected strains, these strains were further tested with the sub-MIC level of OH venom (50% lower than the MIC level; 0.5 × MIC) in 6 h and 24 h biofilms. As such, crude OH-venom at 0.5 × MIC (0.125 mg/mL) reduced biofilms of PAO1 and PACL (Fig. 1A–C) partly through the down-regulated *algD* and *pslB*, the encoded genes for alginate and PSL, respectively, at 6 or 24 h biofilms (Fig. 1D–G). Alginate component in *Pseudomonas* EPS is controlled by AlgU/MucA complex as the binding of MucA protein on the outer membrane (a negative regulator) with the sigma factor AlgU impeding alginate synthesis (Fig. 2A). Interestingly, the crude OH venom downregulated *algU* (Fig. 2B) and *mucA* (Fig. 2D) in 6 h sessile *P. aeruginosa* PAO1, without effect on PACL (Fig. 2C,E), indicating a decrease in alginate production, possibly through AlgU inhibition in early-stage biofilm in a strain-dependent manner. Meanwhile, the PSL component of *Pseudomonas* EPS is regulated by the *psl* operon with GacSA/RsmA regulators (Fig. 2A), and OH venom downregulated *gacA* in both early (6 h) and mature (24 h) biofilms of *P. aeruginosa* PAO1 and PACL (Fig. 2F–G). Hence, crude OH venom demonstrated the bactericidal impact on planktonic *P. aeruginosa* (Table 1) with anti-biofilm activities partly through downregulated genes in the alginate and PSL component syntheses (Figs. 1 and 2).

### **l**-amino acid oxidase from ***O. hannah*** venom (OH-LAAO) as a potential active substance for antimicrobial and anti-biofilm activities against ***P. aeruginosa***

**Figure 3:**
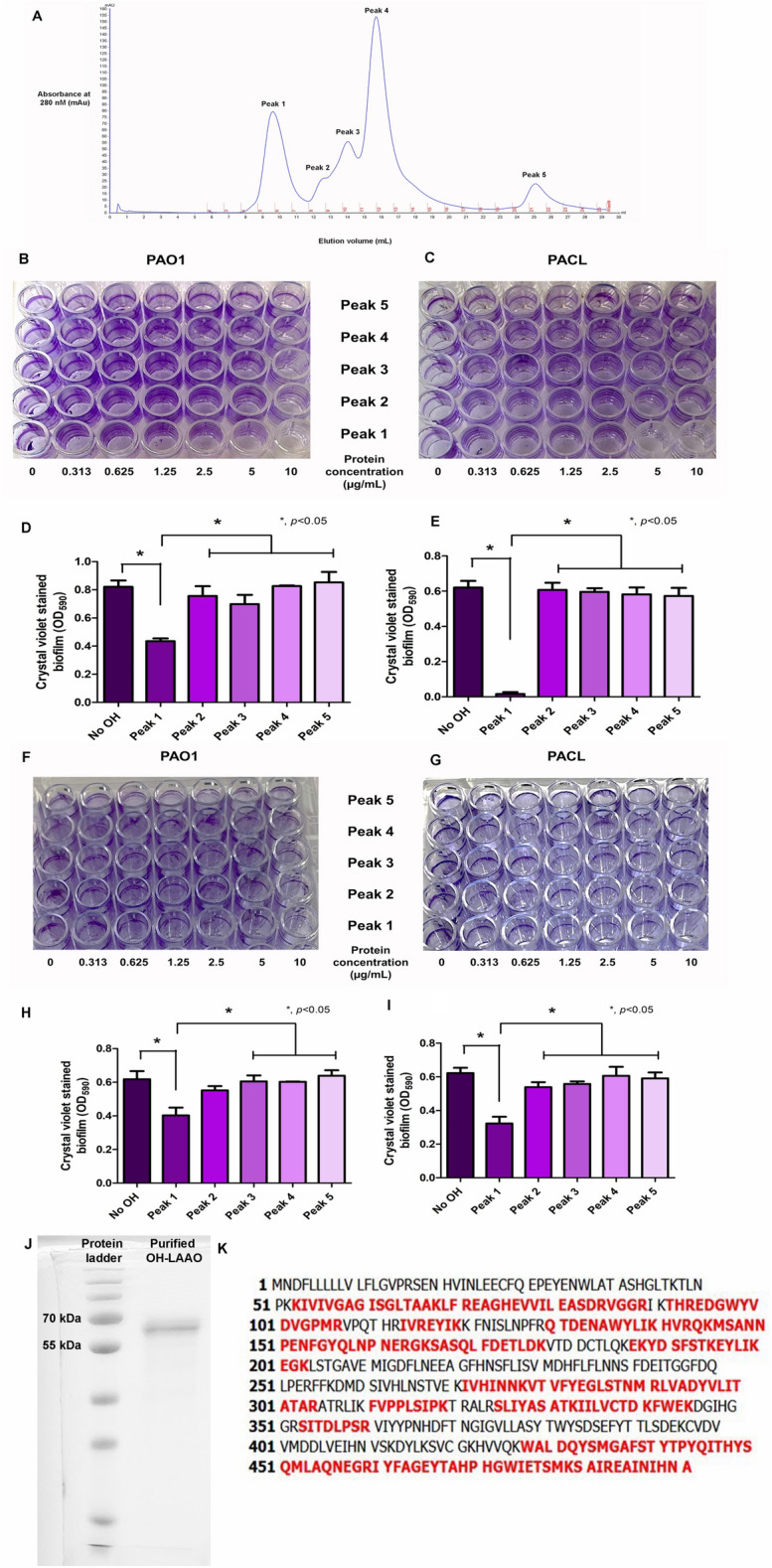
The active peak-1 fraction of crude king cobra venom (OH) with anti-biofilm activity. After the first step of purification using the Sephadex G-75 column chromatography, 5 peaks of crude OH venom are demonstrated, including (**A**). peak-1 (fraction 5–6), peak-2 (fraction 8), peak-3 (fraction 9–10), peak-4 (fraction 11–14), and peak-5 (fraction 20–22) of purified OH venom were collected. The intensities of crystal violet-stained biofilms at 24 h of *P. aeruginosa* PAO1 and *P. aeruginosa* clinically isolated strain (PACL) cultured with each peak of purified OH venoms (5 µg/mL) and the representative 96-well polystyrene plates are demonstrated (**B**–**E**). Likewise, the intensities of crystal violet stain from the 24 h-preformed biofilms of *P. aeruginosa* PAO1 and PACL cultured with each peak of purified OH venoms (10 µg/mL) with the representative 96-well polystyrene plates are demonstrated (**F**–**I**). The experiments were performed in independent triplicate). Mean ± SEM is presented with the one-way ANOVA followed by Tukey’s analysis (*p ˂ 0.05). After purification using a three-step purification (3-column chromatography), the purified OH-LAAO is demonstrated in SDS-PAGE (**J**). The amino acid sequences of purified OH-LAAO (**K**). The results showed 52% coverage of purified OH-LAAO with l-amino-acid oxidase of *O. hannah* (UniProt database: P81383). The matched peptides are demonstrated in red and bold characters.

The crude OH venom was purified by three-step chromatography (see “Methods” Section). The first step was the analytical gel filtration using the Sephadex G-75 column chromatography (Fig. 3A), exploring the effective fractions. Among 5-peaks of the eluted proteins, peak-1 elution demonstrated the most prominent bactericidal activity against *P. aeruginosa* PAO1 and PACL (MIC and MBC at 10 µg/mL) (Table 2) that also showed the most prominent anti-biofilm effect on 24 h biofilms (biofilm prevention experiments) (Fig. 3B–E) and 24 h-established biofilms (biofilm eradication experiments) (Fig. 3F–I), implying biofilm prevention and biofilm detachment properties. These results suggested that antimicrobial, anti-biofilms, and biofilm eradication effects of OH venom were in the peak-1 elution from the first step of purification.

**Table 2:** Antimicrobial, anti-biofilm, and biofilm eradication activities of *Ophiophagus hannah* (OH) elution peak-1 to peak-5 from the first step purification using the Sephadex G-75 column chromatography and purified OH-l-amino acid oxidase (LAAO) from the three-step purification.

Antimicrobial and anti-biofilm parameter (µg/mL)	Bacterial strain	
	*P. aeruginosa* PAO1	*P. aeruginosa* PACL
Gel filtration OH elute peak 1		
MIC	10	10
MBC	10	10
MBIC	10	10
MBEC	> 10	> 10
Gel filtration OH elute peak 2		
MIC	> 10	> 10
MBC	> 10	> 10
MBIC	> 10	> 10
MBEC	> 10	> 10
Gel filtration OH elute peak 3		
MIC	> 10	> 10
MBC	> 10	> 10
MBIC	> 10	> 10
MBEC	> 10	> 10
Gel filtration OH elute peak 4		
MIC	> 10	> 10
MBC	> 10	> 10
MBIC	> 10	> 10
MBEC	> 10	> 10
Gel filtration OH elute peak 5		
MIC	> 10	> 10
MBC	> 10	> 10
MBIC	> 10	> 10
MBEC	> 10	> 10
Purified OH-LAAO		
MIC	5	5
MBC	10	10
MBIC	10	10
MBEC	> 10	> 10

The minimum inhibitory concentrations (MICs) and the minimum bactericidal concentrations (MBCs) were determined for antimicrobials. The minimum biofilm inhibitory concentrations (MBICs) was performed for anti-biofilm activity. The minimum biofilm eradication concentrations (MBECs) was determined for biofilm eradication activity.

Additionally, the specific LAAO activity from these 5 peaks was remarkedly observed only in peak-1 (11.17 ± 1.31 U/mg) (Supplementary Fig. S5). In the second step, the peak-1 elution from the Sephadex G-75 column chromatography was separated and purified for LAAO enzyme using the Resource Q column chromatography, which showed 3 peaks of the elution (Supplementary Fig. S6). The peak-1 eluted from the Resource Q column demonstrated a remarkable LAAO activity at 15.99 ± 1.18 U/mg (Supplementary Fig. S7). The last step of purification used the HiTrap™ Heparin column chromatography (see “Methods” Section), which showed two peaks of the elution (Supplementary Fig. S8). The highest LAAO activity was observed in the peak-1 elution at 21.72 ± 1.39 U/mg (Supplementary Fig. S9). In summary, using the three-step purification provided a significantly higher specific activity of LAAO (Supplementary Fig. S10 and Table S2) for further experiments.

**Figure 4:**
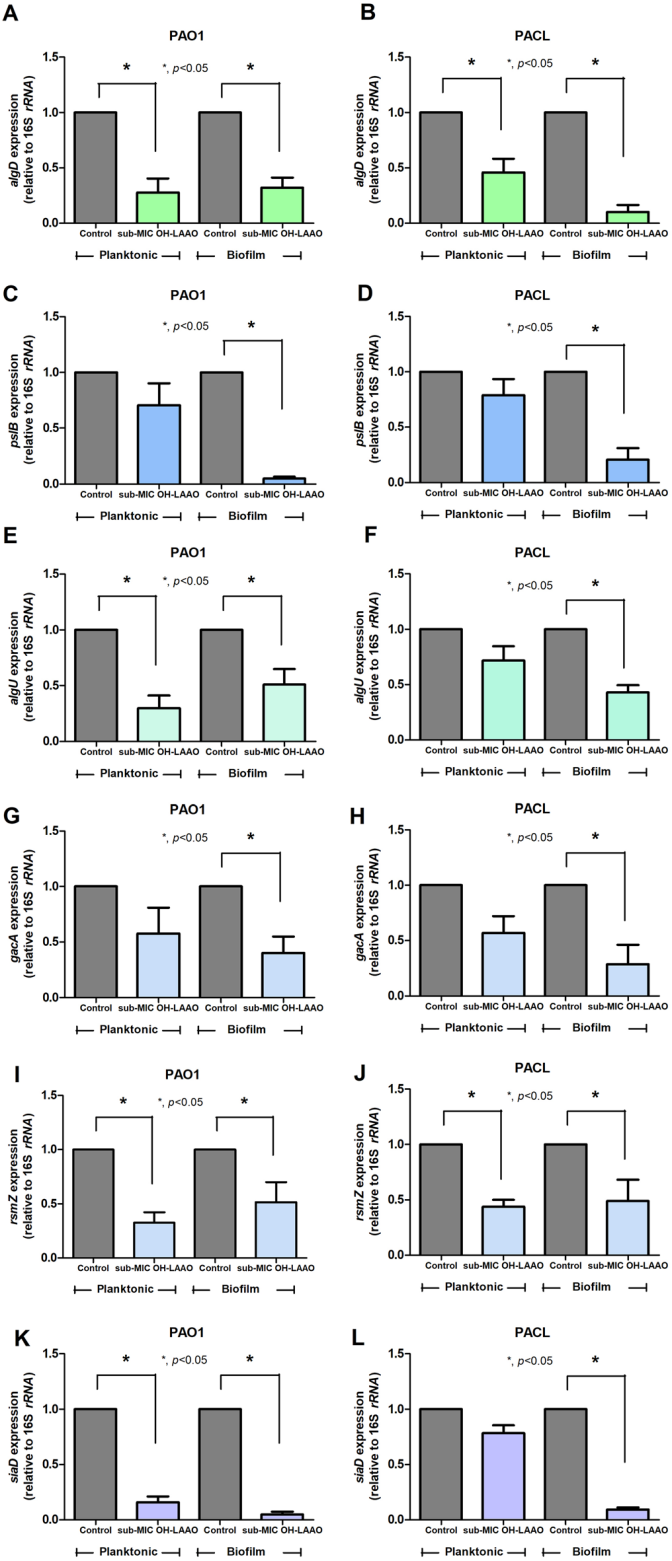
Prevention of biofilm synthesis by l-amino acid oxidase from *O. hannah* venom (OH-LAAO). Gene expression profiles in planktonic (non-biofilm) and sessile (biofilm) forms of *P. aeruginosa* PAO1 and *P. aeruginosa* clinically isolated strain (PACL) biofilms cultured with OH-LAAO (at sub-MIC level, avoiding lethal effect) in alginate (*algD*) (**A**,**B**) and PSL productions (*pslB*) (**C**,**D**), and the regulators, including *algU* (**E**,**F**), *gacA* (**G**,**H**), *rsmZ* (**I**,**J**), and *siaD* (**K**,**L**), as determined by qRT-PCR are demonstrated (the experiments were performed in independent triplicate). Mean ± SEM is presented with the paired *t-*test analysis (*p ˂ 0.05).

Then, an approximately 65 kDa protein dominant in the peak-1 elution from the last step purification using the HiTrap™ Heparin column chromatography (Fig. 3J) was identified by *N*-terminal amino acid sequence using nano-High Performance Liquid Chromatography (nano-HPLC) mass spectrometry and database search (see “Methods” Section). The purified OH-LAAO was matched with LAAO submitted in the UniProt database (UniProt database: P81383) by 52% of LAAO amino acid sequences (Fig. 3K). The purified OH-LAAO (approximately 65 kDa) had a higher molecular weight of LAAO in the database (P81383) (55.977 kDa), indicating the post-translation modification (glycosylation)^21,22^ of OH-LAAO in our study. Therefore, the purified venom from the HiTrap™ Heparin column was further used as OH-derived LAAO (OH-LAAO). After purification, OH-LAAO showed increased antimicrobial activities against *P. aeruginosa* PAO1 and PACL with 5 and 10 µg/mL of MICs and MBCs, respectively (Table 2), and 10 µg/mL of purified OH-LAAO exhibited a prominent anti-biofilm effect (Table 2). Subsequently, the impacts of the purified OH-LAAO against *P. aeruginosa* biofilms were investigated. As such, 2.5 µg/mL of purified OH-LAAO (0.5 × MIC) downregulated *algD* in PAO1 and PACL (planktonic and sessile forms), while *pslB* was downregulated only in sessile but not planktonic cells, and *algU* was downregulated in sessile forms (Fig. 4A–F). Similar to *pslB* downregulation, *gacA*, and *rsmZ*, a small RNA downstream transcribed by the GacA system, were downregulated (Fig. 4G–J). Additionally, expression of *siaD* that encoded diguanylate cyclase for c-di-GMP production, a positive PSL regulator, was depleted in PAO1 and PACL (Fig. 4K–L).

**Figure 5:**
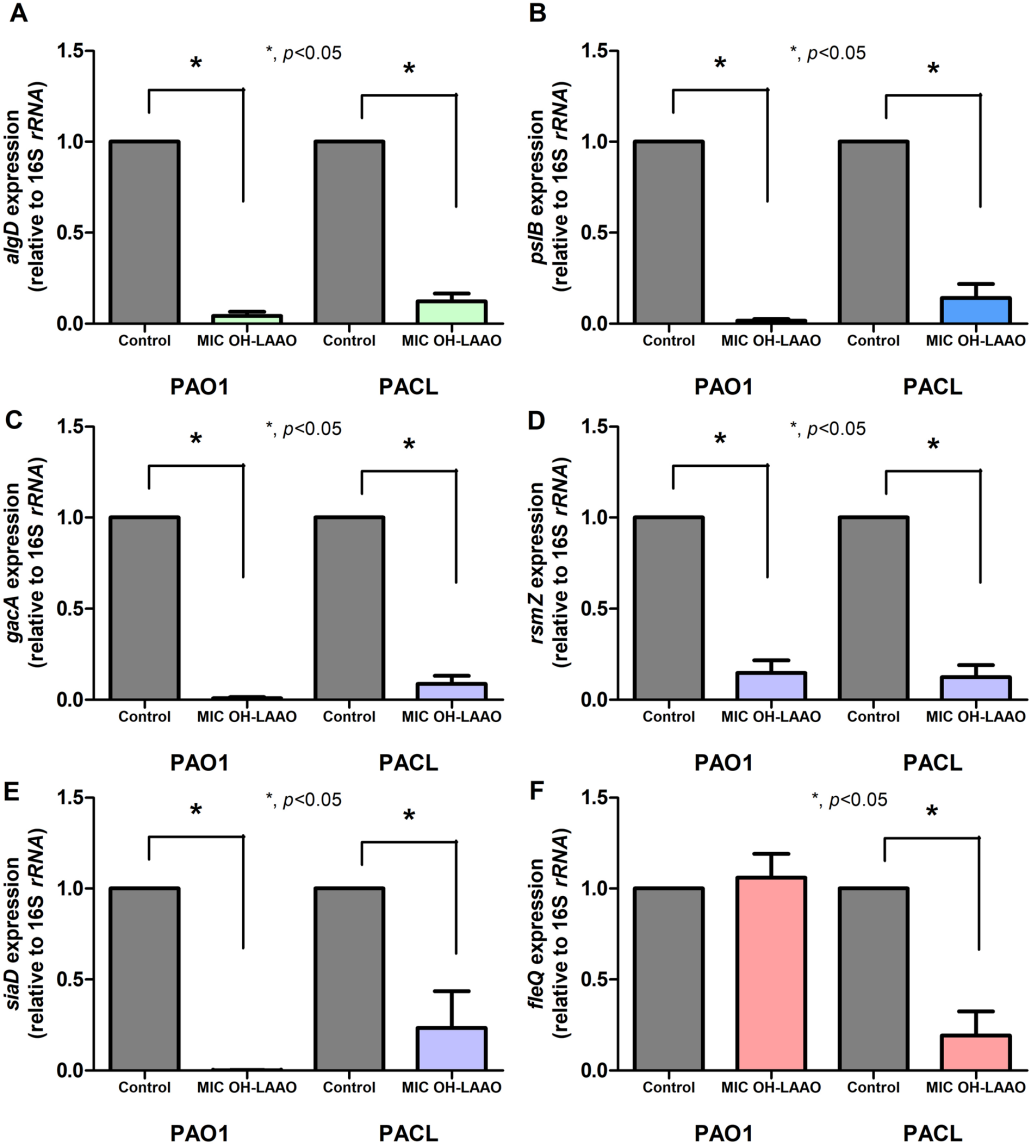
Eradication of the preformed biofilms by l-amino acid oxidase from *O. hannah* venom (OH-LAAO). Gene expression profiles in 24 h preformed biofilms of *P. aeruginosa* PAO1 and *P. aeruginosa* clinically isolated strain (PACL) after incubation with OH-LAAO (at the MIC level) in alginate, *algD* (**A**), and PSL productions *pslB* (**B**), and the regulators, including *gacA* (**C**), *rsmZ* (**D**), *siaD* (**E**), and *fleQ* (an encoding transcriptional regulator controlling gene of flagellum) (**F**), as determined by qRT-PCR are demonstrated (the experiments were performed in independent triplicate). Mean ± SEM is presented with the paired *t-*test analysis (*p ˂ 0.05).

In the biofilm eradication experiments (24 h-preformed biofilms), the purified OH-LAAO downregulated *algD*, *pslB*, *gacA*, *rsmZ*, and *siaD* in both PAO1 and PACL, but downregulated *fleQ* (a flagellar regulator gene) only in PACL (Fig. 5A–F), indicating a possible biofilm erosion through reduced c-di-GMP (on both PAO1 and PACL) and decreased motility (on PACL) as one of the anti-biofilm mechanisms. Therefore, the anti-*Pseudomonas* biofilms of purified OH-LAAO were demonstrated through the reduced synthesis of both alginate and PSL components of biofilms, especially the GacA/*rsmZ* and c-di-GMP-related mechanisms.

### **l**-amino acid oxidase from ***O. hannah*** venom (OH-LAAO) demonstrated antimicrobial and anti-biofilm activities mediated by H_2_O_2_ production

**Figure 6:**
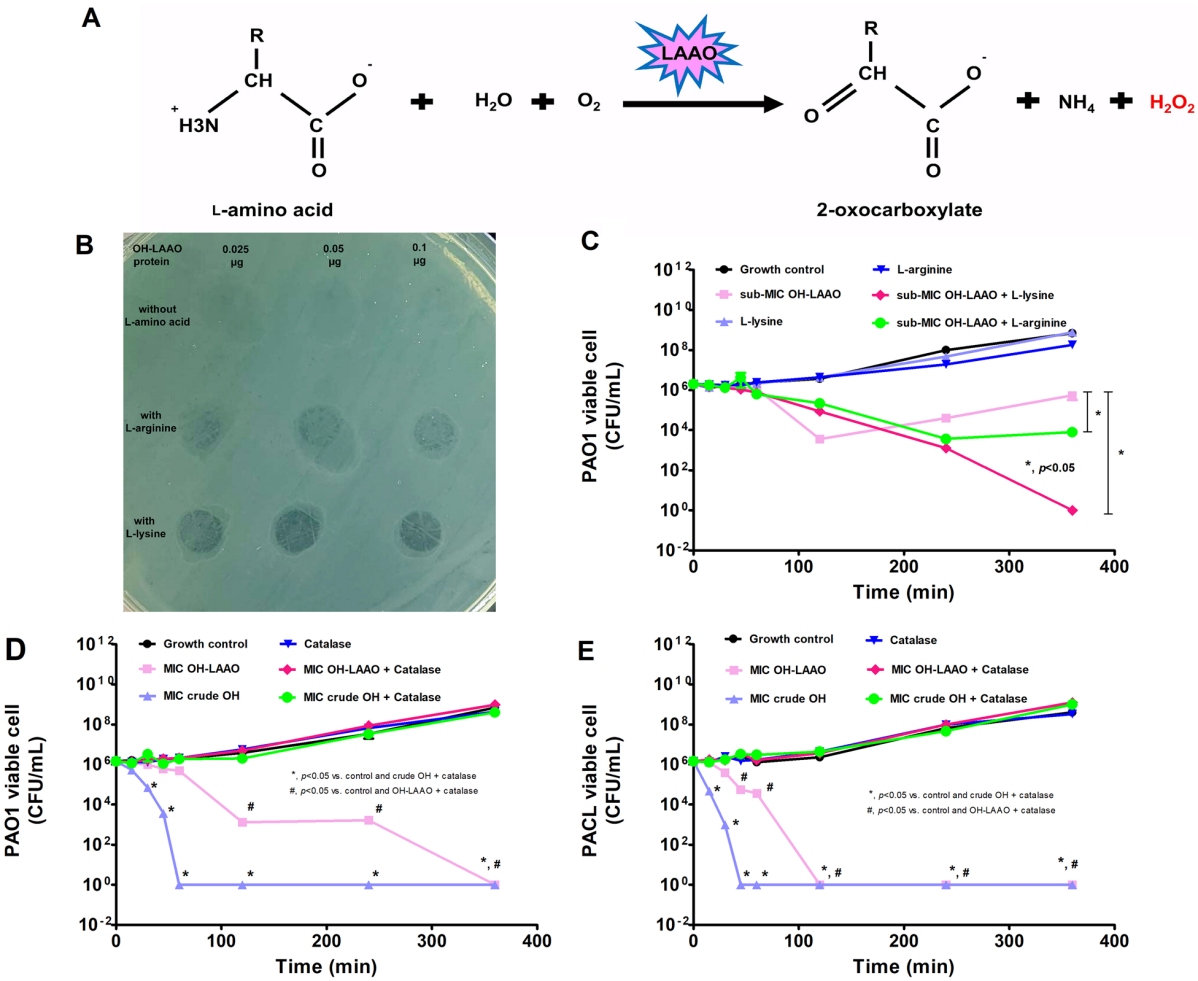
Antimicrobial activity of l-amino acid oxidase from *O. hannah* venom (OH-LAAO), a hydrogen peroxide (H_2_O_2_)-dependent mechanism. Diagram of l-amino acid oxidase (LAAO) enzymatic reaction (**A**), the growth inhibition plate assay (in graph and the representative picture) (**B**,**C**), and the time-kill assay (**D**) using OH-LAAO (0.025–0.1 µg/spot) with or without the substrates (l-arginine and l-lysine at 2.5 mM/spot) against *P. aeruginosa* PAO1 are demonstrated. Also, an effect of catalase, a hydrogen peroxide (H_2_O_2_) decomposer, with either crude OH venom or OH-LAAO in *P. aeruginosa* PAO1 and *P. aeruginosa* clinical isolated strain (PACL) microbicidal activity (**E**) are also indicated. All experiments were performed in triplicate. The data are presented as mean ± SEM with the one-way ANOVA followed by Tukey’s analysis (*p ˂ 0.05 and #*p* < 0.05).

**Figure 7:**
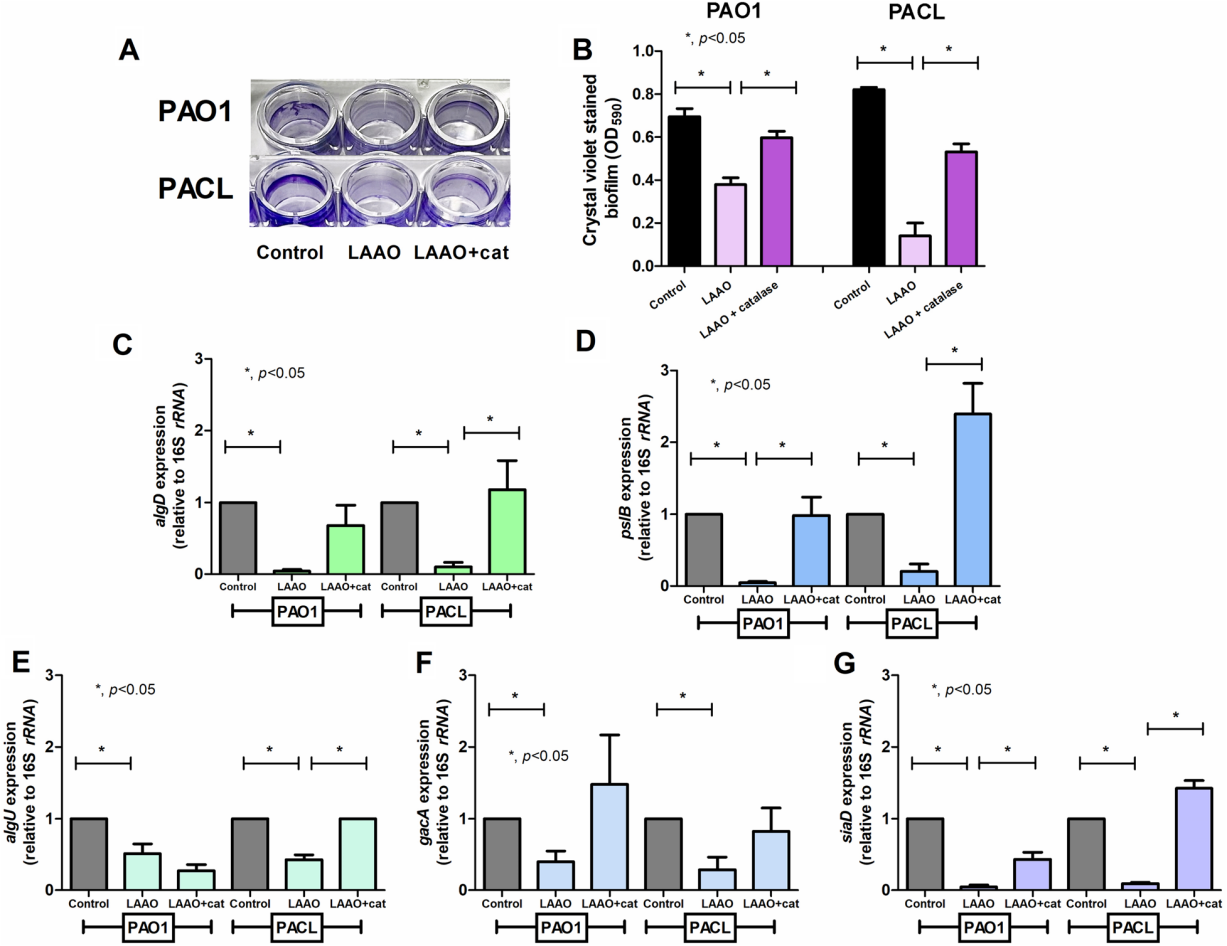
Anti-biofilm activity of l-amino acid oxidase from *O. hannah* venom (OH-LAAO), a hydrogen peroxide (H_2_O_2_)-dependent mechanism. The intensities of crystal violet stain from 24 h biofilms of *P. aeruginosa* PAO1 and *P. aeruginosa* clinically isolated strain (PACL) cultured with OH-LAAO with or without catalase with the representative biofilm pictures in 96-well polystyrene plates stained with crystal violet are demonstrated (**A**,**B**). Gene expression profiles in sessile cells of *P. aeruginosa* PAO1 and PACL cultured with OH-LAAO with or without catalase in alginate (*algD*) (**C**) and PSL productions (*pslB*) (**D**), and the regulators, including *algU* (**E**), *gacA* (**F**), and *siaD* (**G**), determined by qRT-PCR are also demonstrated (the experiments were performed in independent triplicate). Mean ± SEM is presented with the one-way ANOVA followed by Tukey’s analysis (*p ˂ 0.05).

**Figure 8:**
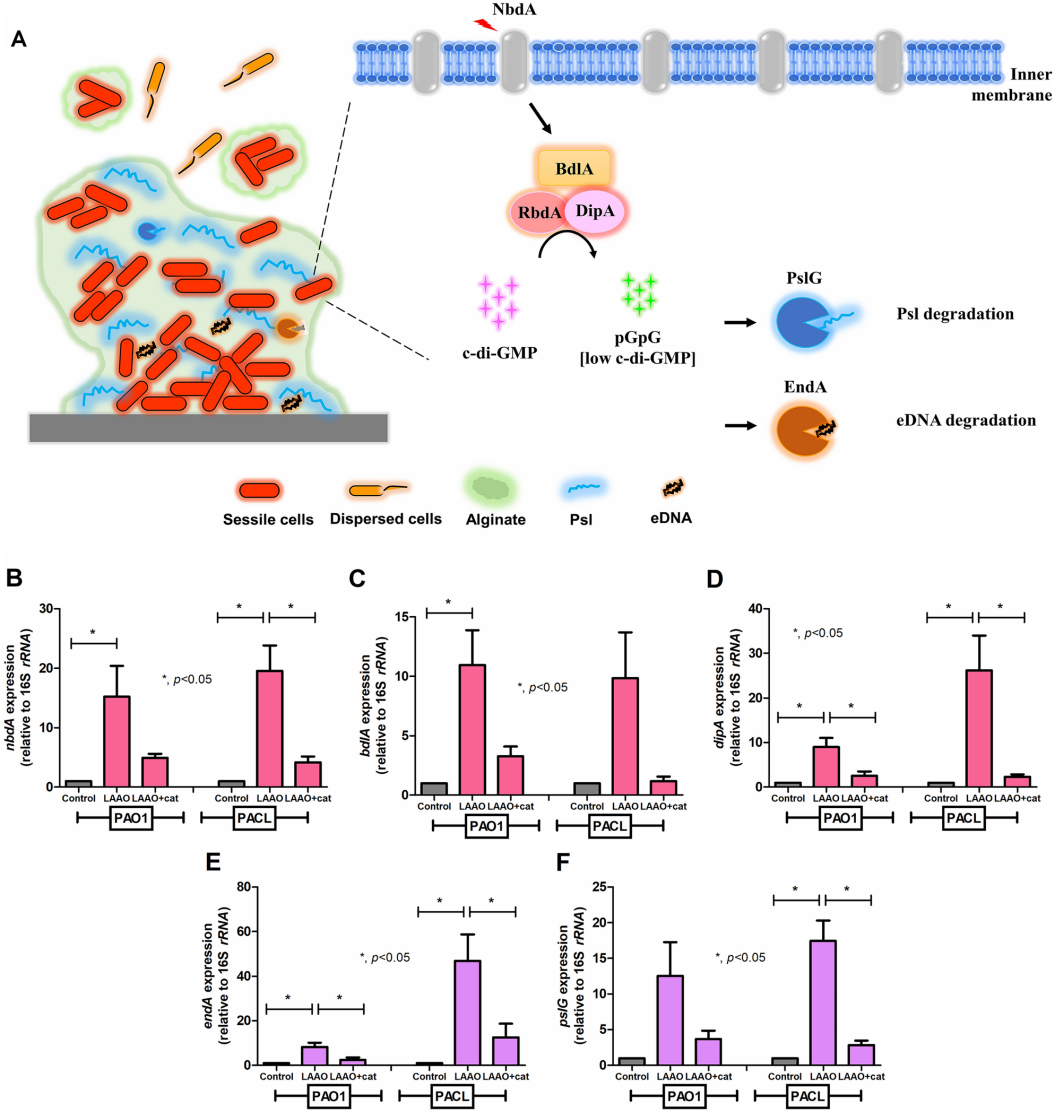
Biofilm dispersion activity of l-amino acid oxidase from *O. hannah* venom (OH-LAAO), a hydrogen peroxide (H_2_O_2_)-dependent mechanism. A diagram of biofilm dispersion in *P. aeruginosa* that external stimulation factors (including nitric oxide; NO) activate NbdA (NO-induced biofilm dispersion) to promote chemotaxis protein (BdlA) that stimulates phosphodiesterase enzymes (DipA and RbdA), resulting in a decreased c-di-GMP level and enhancement biofilm matrix-degrading enzymes (EndA and PslG) (**A**). The expression of biofilm dispersion-related genes, including *nbdA* (**B**), *bdlA* (**C**), and *dipA* (**D**) with the genes of biofilm matrix-degrading enzymes, including *endA* (**E**) and *pslG* (**F**), determined by qRT-PCR, with OH-LAAO incubation with or without catalase on the preformed biofilms of *P. aeruginosa* PAO1 and *P. aeruginosa* clinical isolated strain (PACL) are demonstrated (the experiments were performed in independent triplicate). Mean ± SEM is presented with the one-way ANOVA followed by Tukey’s analysis (*p ˂ 0.05 considered statistically significant).

As one of the hydrolysis enzymes, OH-LAAO induces several metabolites, including 2-oxocarboxylate, ammonium (NH_4_^+^), and hydrogen peroxide (H_2_O_2_), from the substrate (l-amino acids) (Fig. 6A). With the incubation of OH-LAAO with either l-lysine or l-arginine, there was an increased antimicrobial impact against PAO1 planktonic compared to OH-LAAO alone, as indicated by the growth inhibition plate and time-kill assays (Fig. 6B–C). The antimicrobial property of OH-LAAO was neutralized by catalase (an H_2_O_2_ decomposing enzyme) (Fig. 6D–E). Likewise, catalase also neutralized the anti-biofilm effect of OH-LAAO as determined by crystal violet staining and the rescue of several biofilm-associated genes (including *algD*, *pslB*, *algU*, *gacA*, and *siaD*) in PAO1 or PACL with some differences between these strains (Fig. 7A–G). In *P. aeruginosa*, environment sensing proteins (NbdA) recognize several activators, including nitric oxide (NO) and O_2_, that promote several phosphodiesterase enzymes (PDEs) (BdlA and DipA) for c-di-GMP degradation and enhanced biofilm matrix-degrading enzymes, including EndA and PslG, the hydrolyzer against extracellular DNA (eDNA), and PSL hydrolyzer, respectively, resulting in biofilm dispersion (a natural process for the biofilm biomass reduction)^23^ (Fig. 8A). As such, OH-LAAO promoted biofilm dispersion through the enhanced expression of *nbdA*, *bdlA*, *dipA*, *endA*, and *pslG* but was neutralized by catalase (Fig. 8B–F), suggesting a H_2_O_2_-mediated mechanism on NbdA/BdlA/DipA axis (the activation of EndA and PslG; matrix-degrading enzymes). These data implied a crucial role of the enzyme metabolites, especially H_2_O_2_, in the antimicrobial and anti-biofilm properties of OH-LAAO.

## Discussion

### Antimicrobial and anti-biofilm of **l**-amino acid oxidase from ***O. hannah*** venom (OH-LAAO) mediated by H_2_O_2_ metabolite against ***P. aeruginosa*** biofilms

The composition of crude snake venoms depends on species (interspecies variation)^2,24^ and geological habitat (intraspecies variation)^2,25^ as 3FTx of king cobra venom (OH) in Indonesia and Malaysia is 60% and 31% of the substances, respectively^2,26^, while PLA2 of Malaysian and Thai cobra (NK) is 24%, and 16% of the total compounds, respectively^2,26,27^. Hence, the isolation or synthesis of the active molecules from snake venoms can overcome this variation^2,28^. Here, *E. coli* and *E. faecalis* were resistant to most venoms^29,30^, as these bacteria can be isolated from venoms^31^ and some snakebite wounds^32^, indicating a possible natural resistance from the microbial evolution^33^. However, *P. aeruginosa*, the minor microbiota in snake oral cavity^34^ with biofilm production property^10^ and pathogenicity^35^, was susceptible to several snake venoms, especially king cobra venoms (OH). Although both OH and TA venoms exhibited broad spectrum antimicrobial activities as indicated by the MICs and MBCs (Table 1), OH venom had a more prominent eradication against *Pseudomonas* biofilms (Supplementary Figs. S1–S4). Although the venoms from all selected snakes and the purified OH-LAAO could not entirely eradicate biofilms as indicated by the high MBEC levels (Tables 1 and 2), the venoms and OH-LAAO demonstrated some impacts on biofilms as indicated by the acceptable MBIC values.

Indeed, l-amino acid oxidase (LAAOs), an enzyme produced by many living organisms^36^, was identified as a potentially effective compound of OH venom with antimicrobial and anti-biofilm properties, partly through hydrogen peroxide (H_2_O_2_) production. Although LAAOs have various l-amino acid substrates, especially l-leucine^37,38^, LAAO from the king cobra (OH-LAAO) seems to be very specific to l-lysine and l-arginine^39,40^. Indeed, snake LAAOs rapidly and strongly produce more H_2_O_2_ than human LAAOs (encoded by interleukin-4-induced gene 1, *IL4I1*), but the human LAAOs induced higher indole-3-pyruvate (I3P) that promotes cancer survival^41^. Due to the differences in LAAOs between snakes and other hosts, l-lysine and l-arginine which were possibly more specific to OH-LAAO were used in this study. Despite H_2_O_2_ neutralization by several anti-oxidants in most of the living cells^42^, LAAO from venomous animals produces high H_2_O_2_ to induce damage in most cells^36,43^. Because H_2_O_2_-mediated cell surface injury can overcome gene-mediated antibiotic resistance, engineering LAAOs specific to bacterial cells are interesting for combating pan-drug-resistant bacteria, especially against *P. aeruginosa*, with a less adverse effect on the host’s cells. Notably, sub-lethal OH-LAAO was used in our anti-biofilm experiments to avoid interference from microbicidal activity, then the demonstrable anti-biofilm property should be a direct effect on anti-biofilms, but not a simple antimicrobial activity. Although several sub-lethal stress (oxidative stress, H_2_O_2_, and ultraviolet A) can also enhance alginate and PSL production in *P. aeruginosa* biofilms^44,45^, H_2_O_2_ levels from OH-LAAO might be high enough to induce bacterial adverse effects. Likewise, LAAO benefits on *Pseudomonas* biofilms are also supported by Escapin (LAAO derived from the ink of sea hares) through H_2_O_2_ generation with several intermediate products^46^.

### **l**-amino acid oxidase from ***O. hannah*** venom (OH-LAAO) versus several gene-associated ***P. aeruginosa*** biofilms

**Figure 9:**
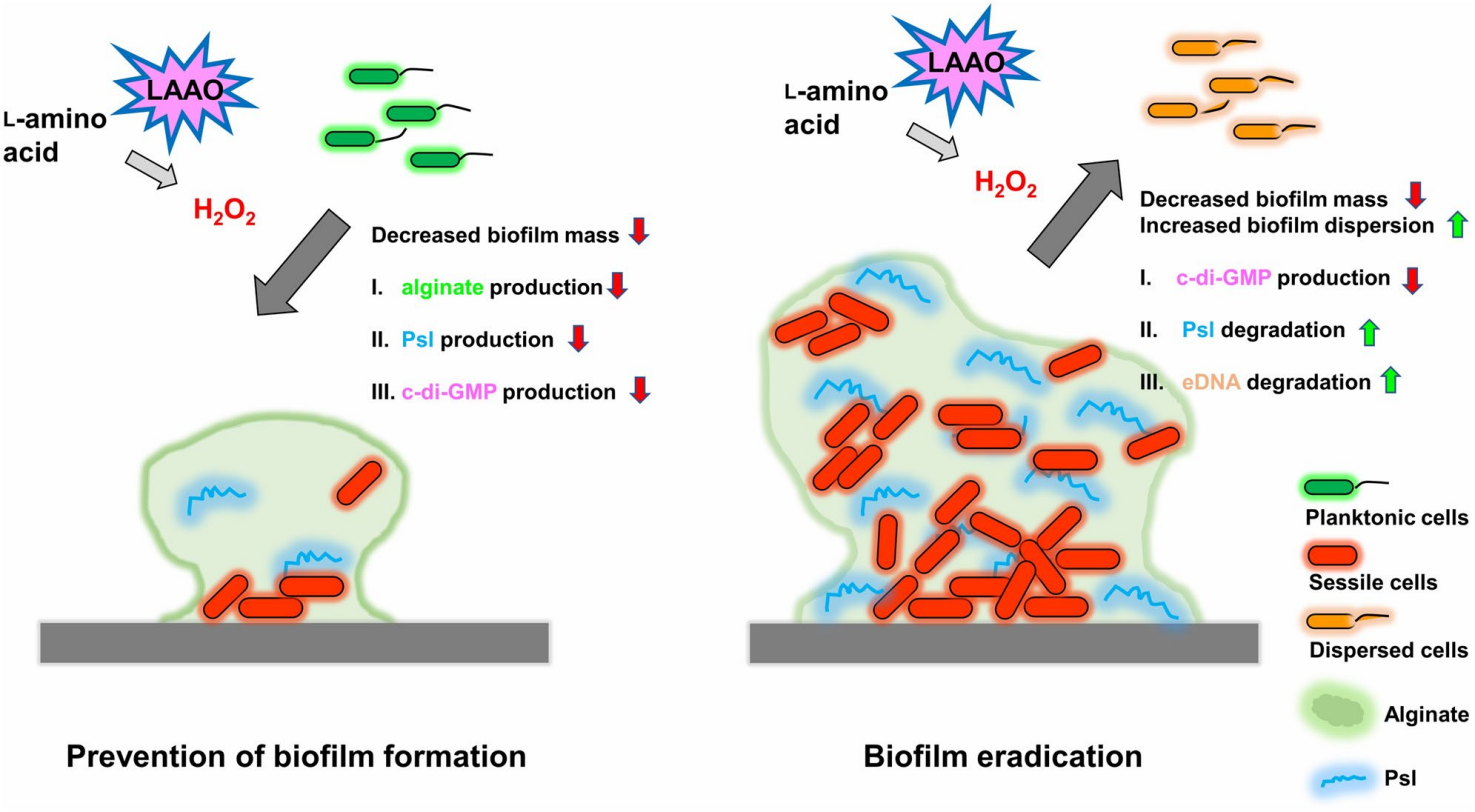
A proposed diagram of hydrogen peroxide (H_2_O_2_)-mediated anti-biofilm and biofilm eradication of l-amino acid oxidase from *O. hannah* venom (OH-LAAO). H_2_O_2_, a metabolite of OH-LAAO, plays a potent role in anti-biofilm against *P. aeruginosa* by impairment of biofilm matrix production (including alginate and PSL) and reduced c-di-GMP levels, resulting in weakened biofilm formation (left side) during the production. For the biofilm eradication (an impact on the preformed biofilms), H_2_O_2_ triggers *P. aeruginosa* biofilm dispersion proteins that alleviate c-di-GMP levels and enhance biofilm matrix-degrading enzymes to actively destroy biofilms (right side).

In harsh environments, *Pseudomonas* biofilm synthesis is initiated by i) alginate synthesis through *alg* operon expression from the release of sigma factor (*algU*) from *mucA* (an *algU* negative regulator) and ii) PSL production from *psl* operon activation (a c-di-GMP dependent process) through diguanylate cyclase (*siaD*) and *gacA* (transcriptional regulator)/*rsmZ* (non-coding small RNA) pathway^47,48^. Here, OH-LAAO inhibited biofilm production in H_2_O_2_ dependent manner, and the effect was neutralized by catalase (an H_2_O_2_ neutralizing enzyme) as concluded in Fig. 9 (left side). Moreover, OH-LAAO attenuated the 24 h pre-formed biofilm, partly through induced biofilm dispersion (the reduced biofilm biomass through the conversion from sessile into planktonic bacteria that are separated from the biofilms^23^). The biofilm dispersion is initiated by *nbdA* (activated regulatory sensor) that triggers phosphodiesterases (*bdlA* and *dipA*) to digest c-di-GMP for inducing degradation of extracellular DNA (eDNA) and PSL (the important biofilm components) through endonuclease A and matrix-degrading enzyme (*pslG*), respectively^23^. Indeed, OH-LAAO promoted biofilm dispersion (Fig. 8), which was neutralized by catalase, suggesting a H_2_O_2_-dependent mechanism, as concluded in Fig. 9 (right side).

Nevertheless, the limitations of biofilm-degrading enzymatic treatment are a necessity of multiple enzymes due to numerous types EPS in biofilms from different organisms and different EPS compositions in each step of biofilm^16^. Interestingly, H_2_O_2_ alone from OH-LAAO suppressed several EPS genes (*algD* and *pslB*) and enhanced numerous EPS-degrading enzymes (*endA* and *pslG*) without a necessity for enzyme combination suggesting the potentially effective biofilm eradication. Although spreading of the infections through the dispersed cells from biofilm^23^, the OH-LAAO antimicrobial activity might reduce this concern. Despite the necessity for further in vivo experiments, OH-LAAO might be useful for the prevention and treatment of biofilms in catheters (catheter-lock solution).

In conclusion, antimicrobial and anti-biofilm activities of crude OH venoms against *P. aeruginosa* were possibly mainly due to LAAO that promoted H_2_O_2_-associated down-regulation of several genes for biofilm production. Moreover, H_2_O_2_ generated by LAAO of OH venom had biofilm eradication activity via increased expressions of several genes for biofilm dispersion. The extraction or synthesis of OH-LAAO is a clinically fascinating candidate for anti-biofilms against *Pseudomonas* spp. More studies are interesting.

## Methods

### Bacterial strains and crude snake venoms

The clinically isolated strains of *P. aeruginosa* (PACL), *S. aureus* (SA1, SA2, and SA3), and *E. faecalis* (EF1, EF2, and EF3) were isolated from the patients in the King Chulalongkorn Memorial Hospital, Bangkok, Thailand. All methods and experimental protocols were approved by the Institutional Review Board of the Faculty of Medicine, Chulalongkorn University (IRB 610/2564) in accordance with the guidelines of the Declaration of Helsinki, and the Institutional Biosafety Committee (MDCU-IBC001/2022) (the biosafety guidelines, Chulalongkorn University) with the waived informed consent (IRB 610/2564). In parallel, chlorhexidine digluconate-treated PACL (C_PACL), a representative *P. aeruginosa* with the enhanced biofilms from an external stimulation (an anti-septic)^49^ and the commercially available bacteria, including *P. aeruginosa* ATCC 27853 and PAO1, *E. coli* ATCC 25922, and *S. aureus* ATCC 29213 were also used.

On the other hand, crude freeze-dried venoms isolated from *N. kaouthia* (NK), *O. hannah* (OH), *D. russellii* (DR), and *T. albolabris* (TA) were obtained from the Queen Saovabha Memorial Institute, Thai Red Cross Society, Bangkok, Thailand.

### l-amino acid oxidase (LAAO) purification from OH venom

The OH crude venom was purified by a three-step procedure of column chromatography using the ÄKTA purification machine (GE Healthcare Life Sciences, Solingen, Germany) to identify the potent fraction against *P. aeruginosa*. Firstly, 200 mg of crude OH venom, 20 mg/mL in 10 mM phosphate buffer saline (PBS) pH 7.3, was loaded to the Sephadex G-75 column (3.2 cm × 150 cm) (GE Healthcare Life Sciences), eluted with 10 mM PBS, and measured for protein concentration by the UV absorbance (*A*_280 nm_; mAU). The fractions belonging to the same *A*_280 nm_ peak were pooled, including peak-1 (fraction 5–6), peak-2 (fraction 8), peak-3 (fraction 9–10), peak-4 (fraction 11–14), and peak-5 (fraction 20–22). All peaks were determined for LAAO activity (mentioned later). Notably, peak-1 from the first step demonstrated the most positive specific LAAO activity among 5 peaks at 11.17 ± 1.31 U/mg. Secondly, the peak-1 elution from the Sephadex G-75 column chromatography was concentrated and dialyzed against 50 mM Tris–HCl, pH 8.0. The concentrated peak-1 elution (6 mL) was subsequently purified using the Resource Q column (1 mL column) (GE Healthcare Life Science), which was equilibrated with 50 mM Tris–HCl, pH 8.0, and eluted with a linear gradient using 0–1 M sodium chloride (NaCl) and 50 mM Tris–HCl, pH 8.0 before measuring protein concentration (*A*_280 nm_). The Resource Q column fractions that were belong to the same *A*_280 nm_ peak, including peak-1 (fraction 12–13), peak-2 (fraction 14), and peak-3 (fraction 15–17), were pooled for LAAO activity determination (mentioned later). The peak-1 eluted from the Resource Q column chromatography (the second step) showed the highest specific LAAO activity among these three peaks at 15.99 ± 1.22 U/mg. Thirdly, the peak-1 from Resource Q column chromatography was concentrated and dialyzed with 50 mM Tris–HCl, pH 8.0 for further purification (the third step purification). Briefly, 5 mL of the concentrated peak-1 was loaded to the equilibrated HiTrap™ Heparin affinity chromatography column (1.6 cm × 2.5 cm, 5 mL) (GE Healthcare Life Science) with 50 mM Tris–HCl (pH 8.0), and eluted with a linear gradient using 0–1 M NaCl with 50 mM Tris–HCl, pH 8.0. Proteins in eluted fractions were measured by *A*_280 nm_. Two peaks of *A*_280 nm_, including peak-1 (fraction 3–7) and peak-2 (fraction 20–22), were observed and collected for LAAO activity assay (mentioned below). Between the peak-1 and peak-2 from the third step purification, the peak-1 showed the greatest specific LAAO activity (21.72 ± 1.39 U/mg) and was used for further experiments as “purified OH-LAAO”.

### l-amino acid oxidase (LAAO) activity test

The crude OH venom and the purified fractions (mentioned above) were determined for LAAO activity using l-leucine as a substrate in the standard enzymatic assay as previously described^50,51^ with a slight modification. Briefly, 0.5 mL of the substrate solution containing 200 mM triethanolamine solution (Sigma-Aldrich, MA, USA), 0.4 mM l-leucine (Sigma Chemical, MO, USA), and 0.0065% *O*-dianisidine (Sigma-Aldrich) was added with 25 µl of 0.007% horseradish peroxidase (Sigma-Aldrich) and 50 µl of the samples to start the enzymatic reaction. The substrate oxidative activity was measured by the increased absorbance at 436 nm (*A*_436 nm_) every 30 s for 3 min. One unit of LAAO enzyme activity was defined as the oxidation of 1 µmole of l-leucine per min. Protein concentrations of the samples were determined by Bradford assay (Thermo Fisher Scientific, MA, USA) with the standard protein using bovine serum albumin (BSA, Sigma-Aldrich). The LAAO enzyme activity was calculated using the following equation:

$$LAAO\,enzyme\,activity \left( {\text{U/mL}} \right) = \frac{{\Delta A436\,nm \times total\,volumn\,of\,the\,assay \left( {{\text{mL}}} \right)}}{{\varepsilon \times volumn\,of\,the\,sample \left( {{\text{mL}}} \right)}}$$

(Δ*A*_436 nm_: the increased absorbance at 436 nm (*A*_436 nm_) per minute, ε: Molecular extinction coefficient of the LAAO reaction product).

The specific LAAO activity was calculated using the following equation:

$$Specific\,LAAO\,enzyme\,activity \left( {\text{U/mg}} \right) = \frac{{LAAO\,enzyme\,activity \left( {\text{U/mL}} \right)}}{{the\,amount\,of\,protein \left( {\text{mg/mL}} \right)}}.$$

### Identification of the isolated l-amino acid oxidase from OH venom (OH-LAAO)

For LAAO identification, the purified fraction from the HiTrap™ heparin column chromatography was separated by sodium dodecyl-sulfate polyacrylamide gel electrophoresis (SDS-PAGE) analysis. Briefly, the proteins (10 µg) were separated by 12% polyacrylamide gel electrophoresis at 100 V for 90 min, followed by staining with Coomassie Brilliant Blue (CBB). After de-staining with Coomassie Brilliant Blue de-staining solution, the gel photo was captured using a camera. Because an approximately 65 kDa protein exhibited a predominated protein band in SDS-PAGE analysis, the protein was processed in the N-terminal amino acid sequence. In brief, the eluted fraction (positive for LAAO activity) from the HiTrap™ Heparin column was treated with 2 µg of trypsin (Sigma-Aldrich, MO, USA) at 37 °C overnight. The tryptic samples were dried in a SpeedVac centrifuge at room temperature, dissolved in 0.1% of trifluoroacetic acid (TFA), loaded onto a 3.5 µm column (Agilent Zorbax 300SB-C18, Agilent Technologies, CA, USA), and eluted with a linear gradient of water/acetonitrile/0.1% formic acid (v/v/v) using the Shimadzu Prominence Nano-High Performance Liquid Chromatography with the electrospray ionization Time-of-flight mass spectrometer (nano-HPLC/ESI-TOF MS, Sciex, MA, USA). The mass spectrometry (MS) data was submitted and analyzed by Mascot sequence matching software (Matrix Science, MA, USA), using the MS data from the UniProt database protein identification.

### Antimicrobial activity of snake venoms

Crude freeze-dried venoms dissolved in sterilized distilled water (DW) were evaluated for antimicrobial activity using the broth microdilution assay. Briefly, 25 µL of crude venoms (20 mg/mL) was added to the final volume of 200 µL of LB broth (Difco, MD, USA) in the 96-well plates, for a maximum concentration of crude venoms at 0.25 mg/mL to avoid the too less of bacterial nourishments in the broth. Then, bacterial isolates (final concentration 1 × 10^5^ CFU/well) were co-cultured in LB broth supplemented with two-fold serial dilutions of crude venoms, followed by incubation at 37 °C for 18 h. The minimal inhibitory concentrations (MICs), defined as the lowest concentration that inhibits bacterial growth, were visually evaluated. Next, the bacterial growth from the broth microdilution method was inoculated on LB agar (Difco) to determine the minimum bactericidal concentrations (MBCs). The MBCs were the lowest venom concentrations that kill bacteria, resulting in no visible growth on LB agar.

### Anti-biofilm activity testing (biofilm prevention)

For biofilm prevention, bacteria (10^8^ CFU/mL) were cultured in 96-well plates containing LB broth supplemented with a serial dilution of either crude venoms or purified OH-LAAO at 37 °C for 24 h. After incubation, the bacterial supernatants were removed by gently pipetting, and the biofilms were washed twice with sterilized distilled water (DW). The biofilms were stained with 0.1% crystal violet (CV) for 15 min and washed twice with DW. The minimum biofilm inhibitory concentrations (MBICs), defined as the lowest concentration that inhibits bacterial biofilm formation, were visually evaluated. The CV-stained biofilms were measured at the OD_590 nm_ (CV detection) to quantify the biofilm masses.

### Biofilm eradication activity testing (experiments on 24 h preformed biofilms)

The biofilm eradication activity of either crude venoms or purified OH-LAAO was tested using 24-h preformed bacterial biofilms. As such, bacteria (10^8^ CFU/mL) were grown in 96-well plates with LB broth at 37 °C for 24 h. Bacterial supernatants (containing bacterial planktonic forms) were removed by aseptically pipetting, and the 24-h biofilms were washed with sterilized DW. The biofilms were treated with LB broth containing either snake venoms or purified OH-LAAO for 24 h at 37 °C. After treatment, the remaining biofilms were stained with 0.1% crystal violet (CV) for 15 min and washed twice with DW. The minimum biofilm eradication concentrations (MBECs), defined as the lowest concentration that completely eradicates bacterial biofilm, were visually evaluated. Moreover, the remaining biofilms were quantified by CV measurement at the OD_590nm_.

### Bacterial gene expressions

The expression of genes encoding for biofilm-related proteins of *P. aeruginosa* was determined by quantitative real-time reverse transcription-polymerase chain reaction (qRT-PCR) using primers listed in Supplementary Table S2. Briefly, the total RNA of planktonic (bacteria without biofilms) or sessile (bacteria with biofilms) forms were extracted by TRIzol^®^ Reagent (Invitrogen, Carlsbad, CA, USA) and was converted to cDNA using RevertAid First Strand cDNA Synthesis Kit (Thermo Scientific, Vilnius, Lithuania). The numbers of gene transcripts were quantified by the QuantStudio6 (Applied Biosystems, Foster City, CA USA) using the SYBR^®^ Green PCR Master Mix (Applied Biosystems). The relative number of transcripts was normalized with *16S rRNA* and calculated using the 2^−ΔΔct^ method.

### OH-LAAO and l-amino acids combination in bacterial inhibition study

Because the substrates of LAAO were l-amino acids, the combination of OH-LAAO and l-amino acids were used against *P. aeruginosa* by the growth inhibition plate assay. Briefly, the lawn of *P. aeruginosa* (10^8^ CFU/mL) on LB agar was spotted by OH-LAAO (0.025–0.1 µg/spot) with or without l-amino acids (l-lysine or l-arginine; Sigma Chemical, MO, USA) (2.5 mM/ spot). After incubation at 37 °C for 24 h, the inhibition zone was visually observed. The increased inhibition of OH-LAAO and l-amino acids was confirmed by the time-kill assay. Briefly, *P. aeruginosa* (10^6^ CFU/mL) was grown in LB broth supplemented with OH-LAAO (at 0.5 × MIC) with or without 250 mM of l-amino acids (l-lysine or l-arginine) at 37 °C with shaking at 120 rpm. The viable bacterial cells at 0, 15, 30, 45, 60, 120, 240, and 360 min were quantified with the drop plate method. Accordingly, bacterial suspensions were diluted serially with sterilized 0.85% NaCl, followed by dropping on LB agar to quantify bacterial colony. Furthermore, catalase, a hydrogen peroxide decomposer, was used in the time-kill study to evaluate if hydrogen peroxide was the potent antibacterial product of OH-LAAO. In brief, *P. aeruginosa* (10^6^ CFU/mL) was grown in LB broth supplemented with snake venoms (either crude OH venom or OH-LAAO) (at 1 × MIC) and catalase (0.1 mg/mL) (SKU, Yiwu, China). The viable bacteria were determined by the drop plate method as described above.

### Statistical analysis

Statistical analysis was performed using the Statistical Package for Social Sciences software (SPSS 22.0, SPSS Inc., IL, USA) and Graph Pad Prism version 7.0 software (La Jolla, CA, USA). All data were presented as mean ± standard error (SE). The differences between two groups and multiple groups were examined for statistical significance by a paired *t*-test or one-way analysis of variance (ANOVA) with Tukey’s analysis, respectively. A *p*-value < 0.05 was considered statistically significant.

## Supplementary Information

Supplementary Information.

## Data Availability

All data generated or analyzed during this study are included in this published article and its supplementary information files.
