# Supplementary material for: Hydrogen peroxide from l-amino acid oxidase of king cobra (Ophiophagus hannah) venom attenuates Pseudomonas biofilms
Source: Sci Rep. 2023 Jul 12;13:11304. doi: 10.1038/s41598-023-37914-3 (PMC10338509; doi:10.1038/s41598-023-37914-3)
Supplement: Supplementary file 1 — Supplementary Information. [file 41598_2023_37914_MOESM1_ESM.pdf]

Hydrogen peroxide from L-amino acid oxidase of king cobra (*Ophiophagus hannah*) venom attenuates *Pseudomonas* biofilms

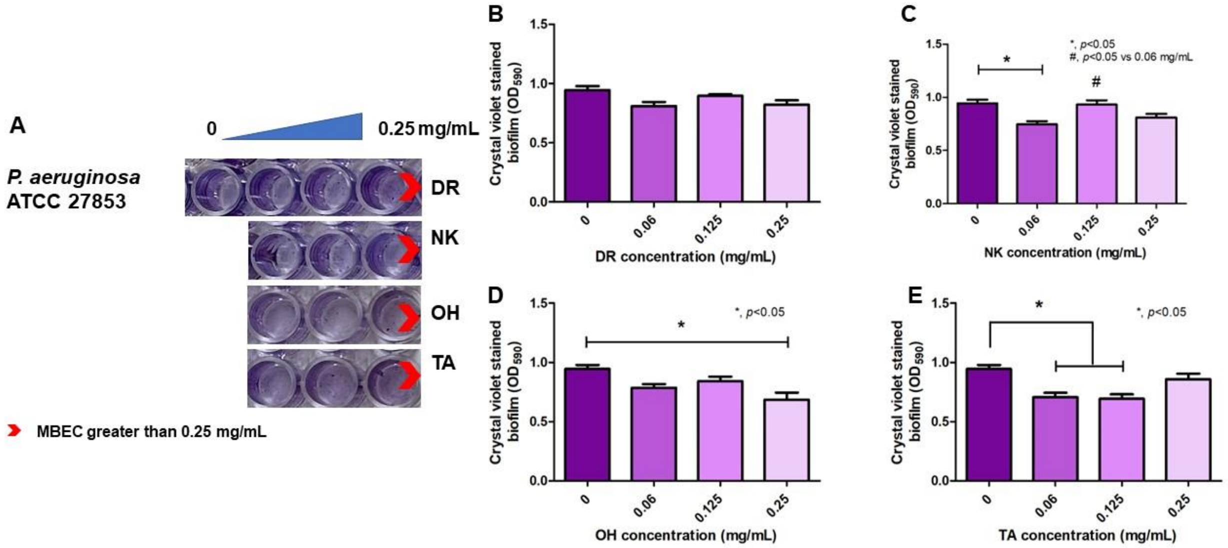

Supplementary Figure S1. Biofilm eradication activity of snake venoms against 24 h-preformed biofilms of *P. aeruginosa* ATCC 27853. The intensity of crystal violet stained remaining biofilms of 24 h preformed biofilms from *P. aeruginosa* ATCC 27853 after eradication by crude venoms of *D. russellii* (DR), *N. kaouthia* (NK), *O. hannah* (OH), and *T. albolabris* (TA) with the representative biofilm pictures in 96-well polystyrene plates stained with crystal violet (A-E) are demonstrated. These experiments were performed in independent triplicate. Mean ± SEM is presented with the one-way ANOVA followed by Tukey's analysis (*, $p<0.05$ and #, $p< 0.05$).

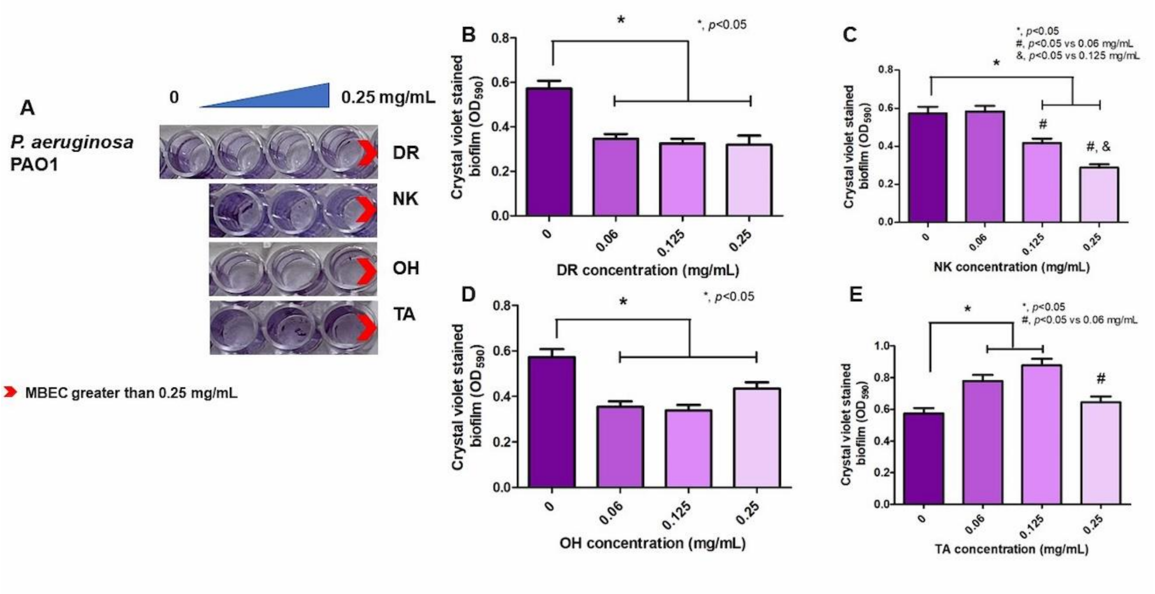

Supplementary Figure S2. Biofilm eradication activity of snake venoms against 24 h preformed biofilms of *P. aeruginosa* PAO1. The intensity of crystal violet stained remaining biofilms of 24 h preformed biofilms from *P. aeruginosa* PAO1 after eradication by crude venoms of *D. russellii* (DR), *N. kaouthia* (NK), *O. hannah* (OH), and *T. albolabris* (TA) with the representative biofilm pictures in 96-well polystyrene plates stained with crystal violet (A-E) are demonstrated. These experiments were performed in independent triplicate. Mean ± SEM is presented with the one-way ANOVA followed by Tukey's analysis (*, p<0.05, $^{\#}$, p< 0.05, and $^{\&}$, p< 0.05).

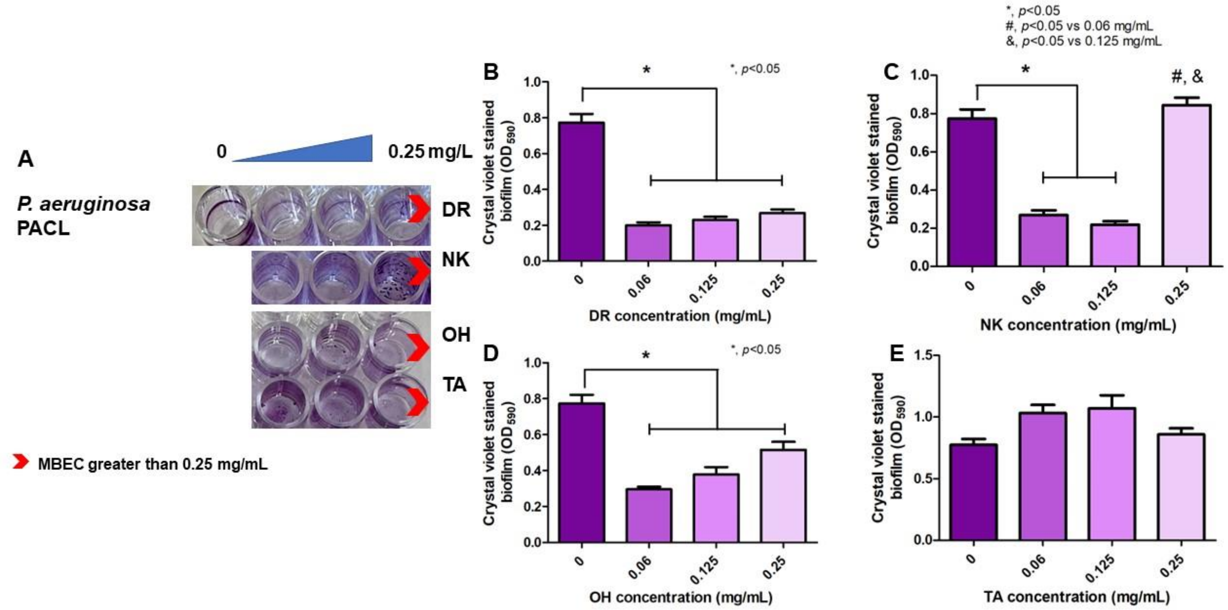

Supplementary Figure S3. Biofilm eradication activity of snake venoms against 24 h preformed biofilms of *P. aeruginosa* PACL. The intensity of crystal violet stained remaining biofilms of 24 h preformed biofilms from clinical isolated *P. aeruginosa* (PACL) after eradication by crude venoms of *D. russellii* (DR), *N. kaouthia* (NK), *O. hannah* (OH), and *T. albolabris* (TA) with the representative biofilm pictures in 96-well polystyrene plates stained with crystal violet (A-E) are demonstrated. These experiments were performed in independent triplicate. Mean ± SEM is presented with the one-way ANOVA followed by Tukey's analysis (*, $p<0.05$, $^{\#}$, $p< 0.05$, and $^{\&}$, $p< 0.05$).

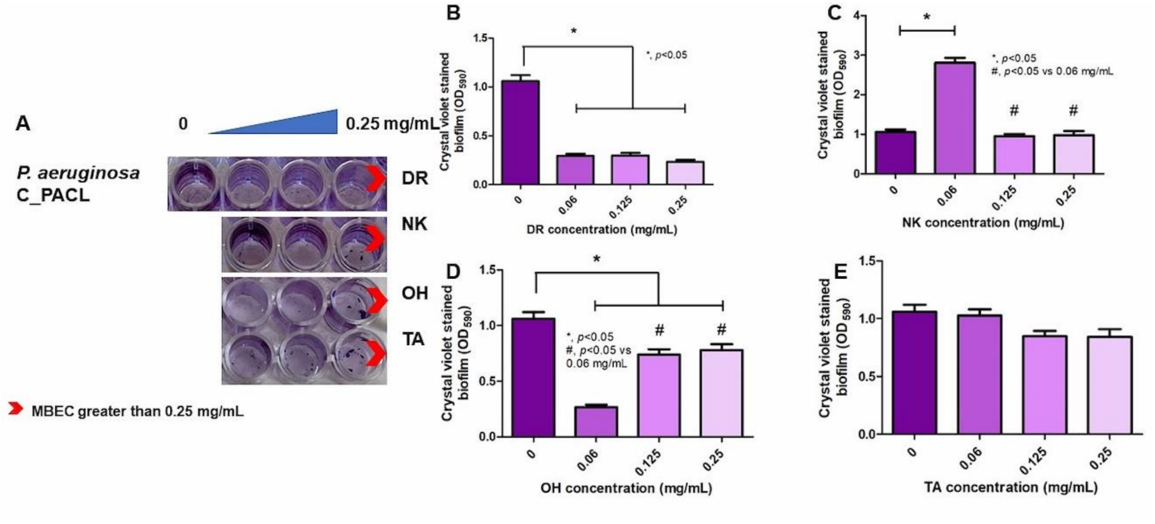

Supplementary Figure S4. Biofilm eradication activity of snake venoms against 24h preformed biofilms of *P. aeruginosa* C_PACL. The intensity of crystal violet stained remaining biofilms of 24 h preformed biofilms from chorhexidine-activated *P. aeruginosa* clinical isolated strain (C_PACL) after eradication by crude venoms of *D. russellii* (DR), *N. kaouthia* (NK), *O. hannah* (OH), and *T. albolabris* (TA) with the representative biofilm pictures in 96-well polystyrene plates stained with crystal violet (A-E) are demonstrated. These experiments were performed in independent triplicate. Mean ± SEM is presented with the one-way ANOVA followed by Tukey's analysis (*, p<0.05 and $^{\#}$, p< 0.05).

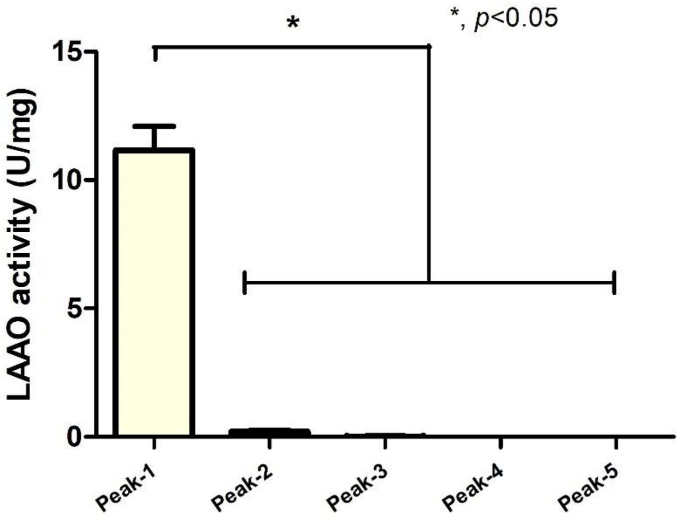

Supplementary Figure S5: L-amino acid oxidase (LAAO) activity of OH venom purified by the first step gel filtration. Crude OH venom was purified by analytical gel filtration using the Sephadex G-75 column. Five peaks (Peak-1 to Peak-5) of purified OH venom were collected (see Methods) and the specific LAAO activity of each peak was determined and calculated to the unit of enzyme (see Methods). These experiments were performed in independent triplicate. Mean ± SEM is presented with the one-way ANOVA followed by Tukey's analysis (*, p<0.05).

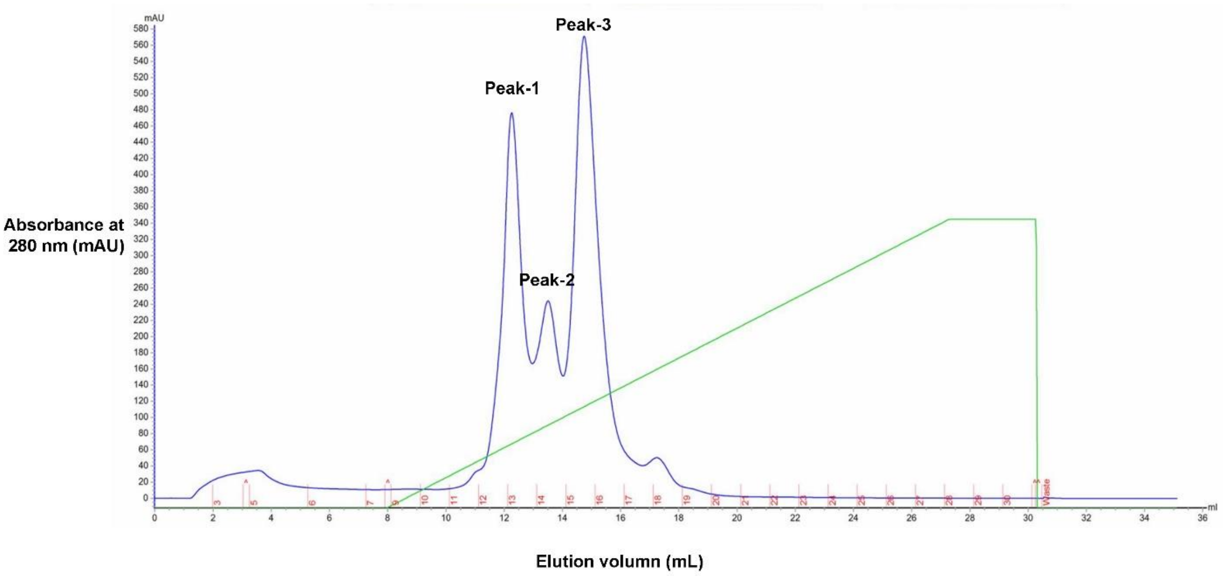

Supplementary Figure S6: The second purification step of OH L-amino acid oxidase (OH-LAAO) using the Resource Q column chromatography. After the first step purification, the Peak-1 elution (positive for LAAO activity) from the Sephadex G-75 column chromatography was performed the second step by the Resource Q ion exchange chromatography. Three major protein fractions were demonstrated and collected, including Peak-1 (fraction 12-13), Peak-2 (fraction 14), and Peak-3 (fraction 15-17).

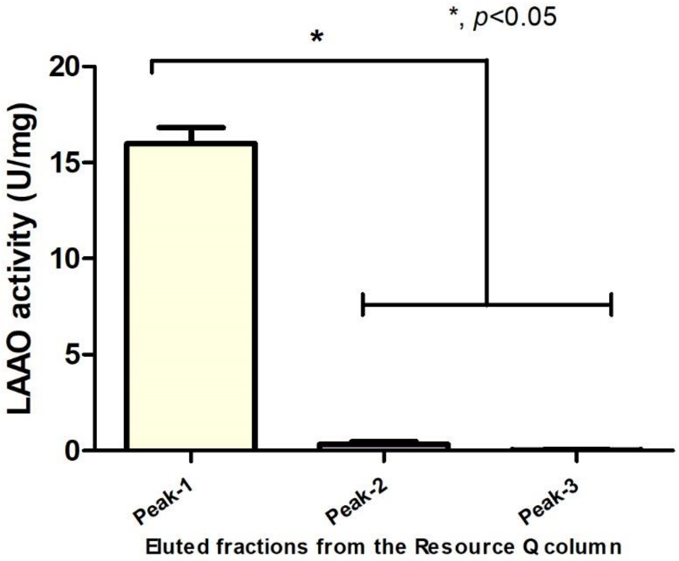

Supplementary Figure S7. L-amino acid oxidase (LAAO) activity of OH venom eluted from the second purification step using the Resource Q ion exchange chromatography. Three major protein fractions, including Peak-1 (fraction 12-13), Peak-2 (fraction 14), and Peak-3 (fraction 15-17) were determined the specific LAAO activity by enzymatic assay (see method). These experiments were performed in independent triplicate. Mean ± SEM is presented with the one-way ANOVA followed by Tukey's analysis (*, $p<0.05$).

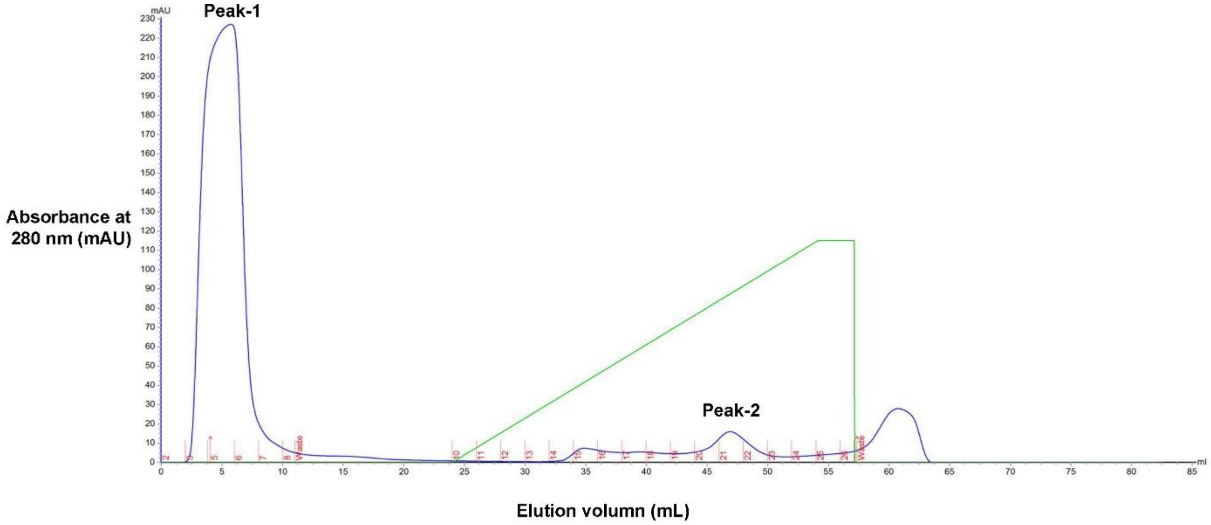

Supplementary Figure S8. The third purication step of OH L-amino acid oxidase (OH-LAAO) using the HiTrap Heparin chromatography. After the second step purification, the Peak-1 elution (positive for LAAO activity) from the Resource Q column chromatography was performed the third step using the HiTrap Heparin chromatography. Two major protein fractions were demonstrated and collected, including Peak-1 (fraction 3-7) and Peak-2 (fraction 20-22).

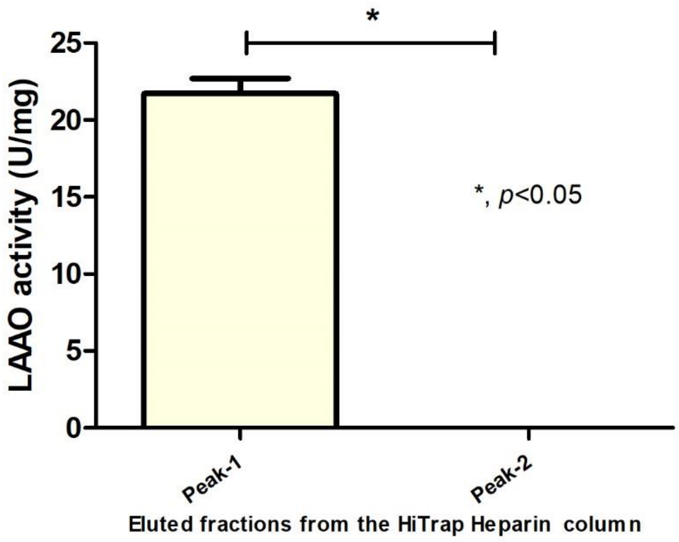

Supplementary Figure S9. L-amino acid oxidase (LAAO) activity of OH venom eluted from the third purification step using the HiTrap Heparin chromatography. Two major protein fractions, including Peak-1 (fraction 3-7) and Peak-2 (fraction 20-22) were determined the specific LAAO activity by enzymatic assay (see method). The Peak-1 showed the strongest specific LAAO activity and used for further investigation as purified OH-LAAO. These experiments were performed in independent triplicate. Mean ± SEM is presented with pair *t*-test analysis (*, $p<0.05$).

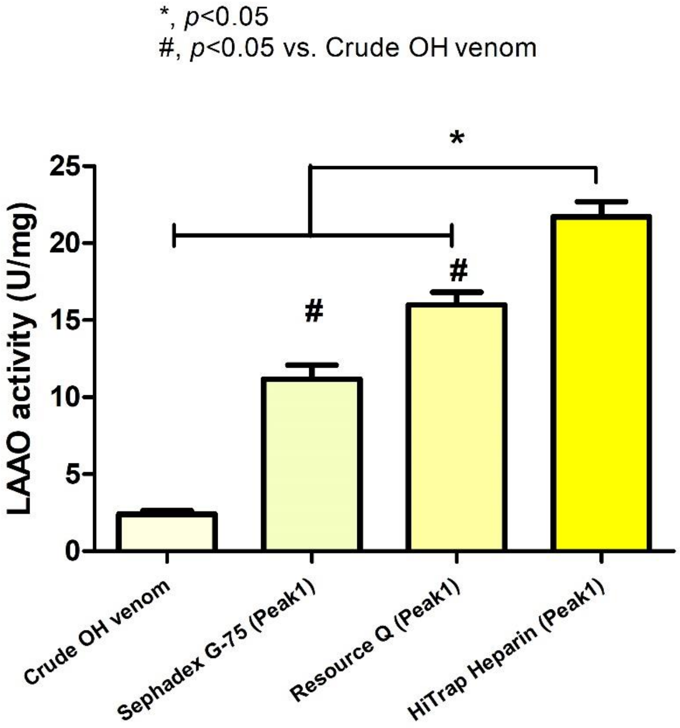

Supplementary Figure S10. The comparation of L-amino acid oxidase (LAAO) activity of crude and purified OH venoms. Crude OH venom and purified OH venoms using the Sephadex G-75 column (Peak-1 sample), the Resource Q column (Peak-1 sample), and the HiTrap Heparin column (Peak-1 sample) were determined for the specific LAAO activity, which was calculated to unit of the enzyme. After the three-step purification, the OH venom showed an significantly increased LAAO activity. These experiments were performed in independent triplicate. Mean ± SEM is presented with the one-way ANOVA followed by Tukey's analysis (*, p<0.05 and $^{\#}$, p< 0.05).

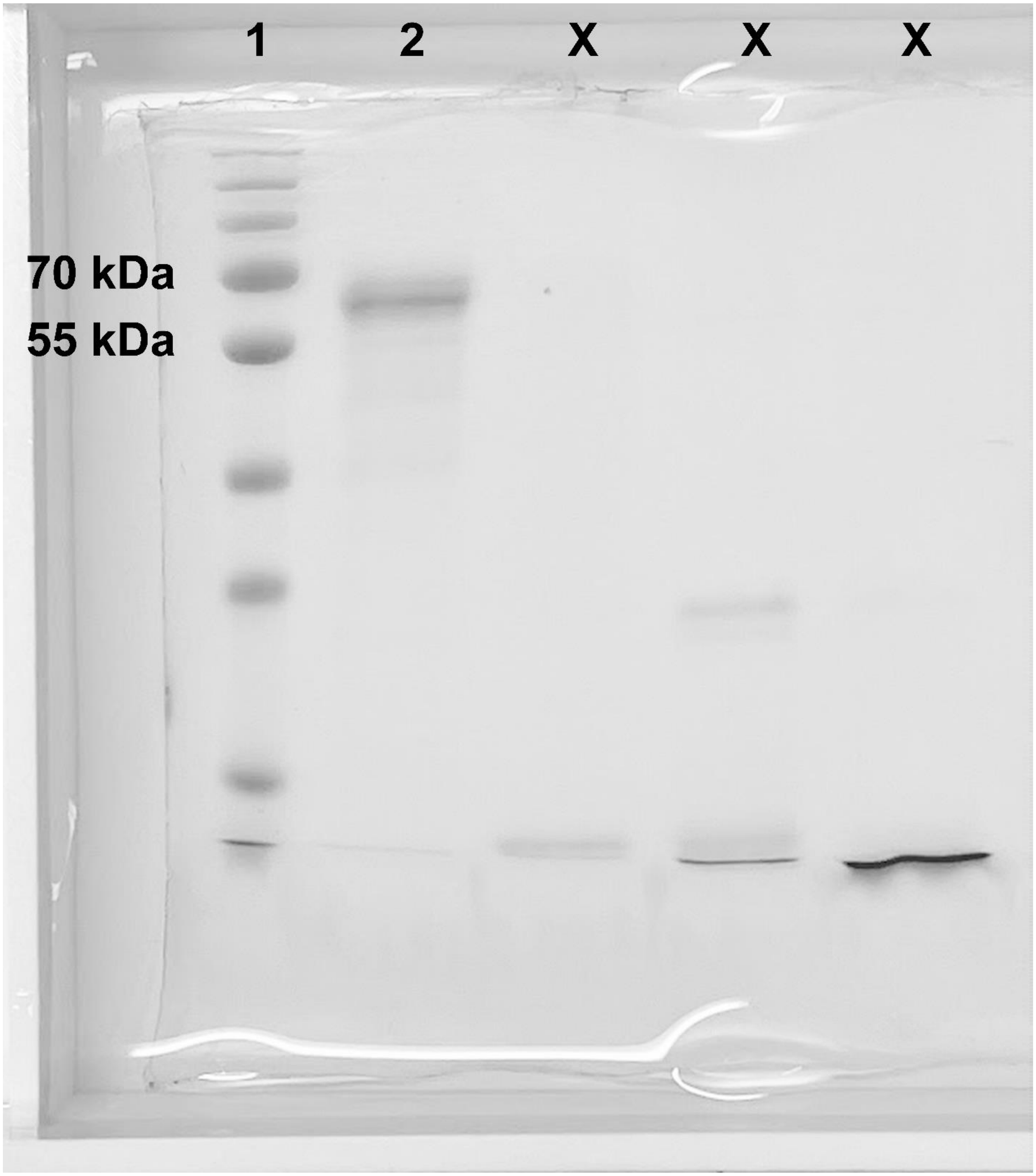

Supplementary Figure S11: Raw image of Figure 3J. Protein contents in the elution from the HiTrap Heparin column using as the purified OH-LAAO were analyzed by SDS-PAGE. The gel photo was captured by a camera. 1: protein ladder, 2: purified OH-LAAO, X: not included in Figure 3J.

Supplementary Table S1: The purification of *O. hannah*-L-amino acid oxidase (OH-LAAO).

Purification step	Protein concentration (mg/mL)	Total protein (mg)	LAAO activity (U/mL)	specific LAAO activity (U/mg)	Purification (fold)	Yield (%)
1. Crude OH venom	20.00	200.00	48.00	2.40	1.00	100.00
2. Peak-1 of the Sephadex G-75 column	1.06	42.50	11.84	11.17	4.65	98.90
3. Peak-1 of the Resource Q column	3.58	21.50	57.24	15.99	6.67	71.62
4. Peak-1 of the HiTrap Heparin column	1.49	13.50	32.54	21.72	9.05	61.09

LAAO enzyme activity was assayed using L-leucine as the substrate.

Supplementary Table S2: Oligonucleotide sequences of primers used in this study.

Gene	Primer sequences (5'-3')	Application	Reference
16S rRNA	ACGCAACTGACGAGTGTGAC	qRT-PCR	1
	GATCGCGACACCGAACTAAT		
algD	GGGCTATGTCGGTGCAGTAT	qRT-PCR	1
	AACGATACGTCGGAGTCCAG		
pslB	GCGAGTTTCTCCTCAACACC	qRT-PCR	2
	CGACCGTAGATGTCGTTGAA		
algU	CAGAGGATGCGGAGTTCTTC	qRT-PCR	1
	ACGGTGGCGATATCTTCGTA		
mucA	CGTGCGTCTGTACAACCAGA	qRT-PCR	1
	CTGGACGAGGAGTTGGTGAT		
gacA	CTATATCAGCCCGCAGATCG	qRT-PCR	2
	CTTCTCGAAGATGCGGTAGC		
rsmZ	CGTACAGGGAACACGCAAC	qRT-PCR	2
	TATTACCCCGCCCACTCTTC		
siaD	GCTACGCGAATACGACCTCT	qRT-PCR	2
	ATTGACGGTCTGCGAATAGG		
fleQ	GGTCTACCGCGAGATGTACG	qRT-PCR	This study
	GGAATGGTAATGCAGGTTGC		
nbdA	AACATCGACCAGACCATCCC	qRT-PCR	This study
	CCATGATGAACGAGCGATCC		
bdlA	ACCTCGATCGTCAACACCAT	qRT-PCR	This study
	ATCATGTCGGCGATCTCCTT		
dipA	AGGTCCTGATGAACCACGAG	qRT-PCR	This study
	ACTGGGTGATCGAGGTGAAG		
endA	GCCCGCACCTACTTCTACAT	qRT-PCR	This study
	AGGATAGGTTGCTGCAGGTT		
pslG	GCCACCTACCACCCCTATAC	qRT-PCR	This study
	GCCAATGATGTCCTGCAACT		

References

1. Phuengmaung, P., Somparn, P., Panpetch, W., Singkham-In, U., Wannigama, D.L., Chatsuwan, T. & Leelahavanichkul, A. Coexistence of *Pseudomonas aeruginosa* with *Candida albicans* enhances biofilm thickness through alginate-related extracellular matrix but is attenuated by N-acetyl-L-cysteine. *Front Cell Infect Microbiol*. **10**, 594336, doi: 10.3389/fcimb.2020.594336 (2020).

2. Singkham-In, U., Phuengmaung, P., Makjaroen, J., Saisorn, W., Bhunyakarnjanarat, T., Chatsuwan, T., Chirathaworn, C., Chancharoenthana, W. & Leelahavanichkul, A. Chlorhexidine promotes Psl expression in *Pseudomonas aeruginosa* that enhances cell aggregation with preserved pathogenicity demonstrates an adaptation against antiseptic. *Int J Mol Sci*. **23**(15), 8308, doi: 10.3390/ijms23158308. (2022).
